# Phylogenetic and chemical diversity of fungal endophytes isolated from *Silybum marianum* (L) Gaertn. (milk thistle)

**DOI:** 10.1080/21501203.2015.1009186

**Published:** 2015-02-23

**Authors:** Huzefa A. Raja, Amninder Kaur, Tamam El-Elimat, Mario Figueroa, Rahul Kumar, Gagan Deep, Rajesh Agarwal, Stanley H. Faeth, Nadja B. Cech, Nicholas H. Oberlies

**Affiliations:** a Department of Chemistry and Biochemistry, University of North Carolina at Greensboro, Greensboro, NC27402, USA; b Facultad de Química, Universidad Nacional Autónoma de México, Mexico, DF04510, Mexico; c Department of Pharmaceutical Sciences, Skaggs School of Pharmacy and Pharmaceutical Sciences, University of Colorado Denver, Aurora, CO80045, USA; d Department of Biology, University of North Carolina at Greensboro, Greensboro, NC27402, USA

**Keywords:** Ascomycota, endophyte, *Silybum marianum*, milk thistle

## Abstract

Use of the herb milk thistle (*Silybum marianum*) is widespread, and its chemistry has been studied for over 50 years. However, milk thistle endophytes have not been studied previously for their fungal and chemical diversity. We examined the fungal endophytes inhabiting this medicinal herb to determine: (1) species composition and phylogenetic diversity of fungal endophytes; (2) chemical diversity of secondary metabolites produced by these organisms; and (3) cytotoxicity of the pure compounds against the human prostate carcinoma (PC-3) cell line. Forty-one fungal isolates were identified from milk thistle comprising 25 operational taxonomic units based on BLAST search via GenBank using published authentic sequences from nuclear ribosomal internal transcribed spacer sequence data. Maximum likelihood analyses of partial 28S rRNA gene showed that these endophytes had phylogenetic affinities to four major classes of Ascomycota, the Dothideomycetes, Sordariomycetes, Eurotiomycetes, and Leotiomycetes. Chemical studies of solid–substrate fermentation cultures led to the isolation of four new natural products. In addition, 58 known secondary metabolites, representing diverse biosynthetic classes, were isolated and characterized using a suite of nuclear magnetic resonance and mass spectrometry techniques. Selected pure compounds were tested against the PC-3 cell line, where six compounds displayed cytotoxicity.

## Introduction

Endophytes are fungi or bacteria that inhabit healthy living plant tissues for all or part of their life cycle without causing disease (Wilson 1995). Fungal endophytes are ubiquitous among plants and are phylogenetically and ecologically diverse (Petrini 1991; Arnold 2001, 2007); they are isolated often by rigorous surface sterilization of healthy plant parts followed by subsequent incubation in nutrient media (Carroll 1991). Two major groups of endophytes can be recognized based on their mode of transmission, the systemic, vertically transmitted endophytes that have been found in grasses (Clavicipitaceae, Ascomycota), and the non-systemic, horizontally transmitted endophytes that have been found in all terrestrial and aquatic plants examined to date (Rodriguez et al. 2009). Together, these fungal endophytes represent an abundance of unexplored and novel diversity, both from mycological (Faeth and Hammon 1997; Saikkonen et al. 1998; Arnold et al. 2000; Arnold 2001, 2007; Suryanarayanan et al. 2005; Saikkonen 2007; Gazis et al. 2012) and chemical perspectives (Strobel and Daisy 2003; Suryanarayanan et al. 2009; Kharwar et al. 2011), thus warranting intensive exploration of these hyperdiverse endosymbionts for phylogenetic and chemical diversity (Smith et al. 2008).

Over the last decade, there have been numerous studies on fungal endophytes of medicinal plants (Tan and Zou 2001; Strobel and Daisy 2003; Kumar and Hyde 2004; Tejesvi et al. 2007, 2011; Huang et al. 2008; Naik et al. 2008; Xu et al. 2010; Chen et al. 2011; de Siqueira et al. 2011; Verma et al. 2011; Bascom-Slack et al. 2012; Miller, Qing, Sze, Neilan, et al. 2012; Miller, Qing, Sze, Roufogalis, et al. 2012; Langenfeld et al. 2013), which have begun to address questions about the diversity and distribution patterns of these endophytes in various parts of the plant. In addition, endophytes have also been investigated in search for new secondary metabolites (Schulz et al. 2002; Chomcheon et al. 2005, 2006; Puri et al. 2005; Gu et al. 2007; Aly et al. 2008, 2011; Kusari et al. 2009; Debbab et al. 2009). However, fungal endophytes from a well-known ethnobotanical plant commonly known as milk thistle, *Silybum marianum* (L.) Gaertn. (Asteraceae), have not been explored, and thus both the fungal and the chemical diversity of endophytes in this herb remain uncharacterized.

Milk thistle consistently ranks among the best-selling herbs in the USA (Blumenthal et al. 2006). Seeds from milk thistle have been used since antiquity for their hepatoprotective properties (Kroll et al. 2007; Polyak et al. 2013). Through several fruitful collaborations, members of our team have been investigating this herb for over a decade including studies for prostate cancer chemoprevention (Davis-Searles et al. 2005; Deep et al. 2007; Deep, Oberlies, et al. 2008; Deep, Raina, et al. 2008; Deep et al. 2010; Graf et al. 2007), treatment of hepatitis C infection (Morishima et al. 2010; Polyak et al. 2010; Wagoner et al. 2010, 2011; Blaising et al. 2013), and to evaluate enteric metabolism (Brantley et al. 2010, 2013, 2014). Phytochemical studies on milk thistle have led to the isolation of seven major diastereoisomers (Kim et al. 2003; Lee and Liu 2003). Although much is known about the phytochemistry of milk thistle, major gaps remain in our knowledge about the fungal species composition and mycochemisty of this plant. Despite chemical investigations of plant-based metabolites of milk thistle that spans >50 years (Sy-Cordero et al. 2012; Napolitano et al. 2013), no studies on the endophytes from this botanical have been reported. In the last year, however, members of our team showed that silybin A, silybin B, and isosilybin A, three of the seven flavonolignans that constitute silymarin, an extract of milk thistle seeds (achenes) (Kroll et al. 2007), were detected for the first time from a fungal endophyte, *Aspergillus iizukae* (G77), which was isolated from the surface-sterilized leaves of milk thistle (El-Elimat et al. 2014). We also reported on a series of polyhydroxyanthraquinones from the guttates of *Penicillium restrictum* (G85), which was isolated from the stems of milk thistle (Figueroa et al. 2014).

To further understand the chemical mycology of fungal endophytes of milk thistle, a culture-based approach was initiated using molecular data from the nuclear ribosomal genes, such as internal transcribed spacer (ITS) region and the partial region of the large subunit (LSU) nrRNA gene, to identify the fungal endophytes of this medicinal herb. The primary goal was to culture fungal endophytes from leaves, stems, roots, and seeds of milk thistle to evaluate species composition and phylogenetic diversity. Additionally, we profiled the chemical diversity by identifying the secondary metabolites produced by these fungal endophytes (Strobel and Strobel 2007; Bascom-Slack et al. 2012). Finally, as part of our efforts towards screening our in-house library of pure compounds in available bioassays, selected metabolites that were obtained in sufficient quantity and purity during the course of these studies were also evaluated for cytotoxicity against a prostate cancer cell line (PC-3).

## Materials and methods

### Sampling of plants

Whole plants and seeds of *Silybum marianum* (L.) Gaertn. (Asteraceae) were obtained from Horizon Herbs, LLC (Williams, OR, USA). A voucher specimen was deposited at the University of North Carolina Herbarium (NCU602014). Four different collections of whole plants and seeds were obtained for the study in 2011 (Lot # 6490; Lot # 6510), 2012 (Lot # 12348), and 2013 (Lot # 6462).

### Isolation of endophytic fungi

Fungal endophytes were isolated from healthy living milk thistle plants. The stems, leaves, roots, and seeds of the plant were cut into small pieces (~2–5 mm in length) and washed with tap water. Subsequently, the segments were surface sterilized by sequential immersion in 95% ethanol (10 s), sodium hypochlorite (10–15% available chlorine, Sigma) (2 min), and 70% ethanol (2 min) using a modification of the protocol described previously (Arnold et al. 2001, 2003; Arnold and Lutzoni 2007). The plant segments were transferred using aseptic conditions onto 2% malt extract agar (MEA, Difco, 20 g MEA, 1 L sterile distilled water amended with antibiotics streptomycin sulphate of 250 mg l^−^^1^ and penicillin G of 250 mg l^−^^1^); antibiotics were used to prevent the growth of bacterial endophytes. To test the efficacy of the surface-sterilization procedure, individual surface-sterilized segments were touched and then removed from the surface on separate 2% MEA plates with antibiotics. The absence of fungal growth on the nutrient media confirmed that the sterilization procedure was effective in eliminating epiphytic fungi (Shultz et al. 1998). A total of 605 segments of leaf, stem, roots, and seeds were plated. Plates were sealed with parafilm and incubated at room temperature (RT) in 12 h of dark/light until the emergence of fungal colonies was observed. The fungi were subsequently grown on 2% MEA, Potato Dextrose Agar (PDA, Difco), and 2% soy peptone, 2% dextrose, and 1% yeast extract (YESD). The fungal cultures are maintained at 9°C at the University of North Carolina at Greensboro, Department of Chemistry and Biochemistry Fungal Culture Collection.

### Characterization of fungal endophytes

Where possible, colony morphospecies identifications were made using methods outlined earlier (Gazis and Chaverri 2010). However, as many fungal endophytes do not sporulate in culture, species identification using morphological characters can be challenging (Arnold and Lutzoni 2007; Hyde and Soytong 2008). Therefore, molecular sequence data from the nuclear ribosomal ITS along with the 5.8s region were used since this region has been designated as an official barcode for fungi (Schoch et al. 2012). In addition, the first two domains of the nuclear ribosomal LSU (nrRNA gene) were sequenced to evaluate phylogenetic diversity and distribution within the Ascomycota (Liu et al. 2012).

### DNA extraction and PCR amplification

For extraction of genomic DNA, mycelium from axenic cultures was scraped with a sterile scalpel from plastic Petri plates and ground to a fine powder in liquid nitrogen using a mortar and pestle. Approximately 400 µl of AP1 buffer from the DNeasy Plant Mini Kit (QIAGEN Inc., Valencia, CA, USA) was added to the mycelial powder, and DNA was extracted following the manufacturer’s instructions. The DNA was eluted in 50 µl of molecular biology grade water. Total genomic DNA was observed on a 1% tris-borate-ethylenediaminetetraacetic acid agarose gel stained with ethidium bromide. Fragments of complete ITS, ~600–650 bp and partial LSU (hypervariable regions D1/D2 divergent domains; ~600 bp) (Liu et al. 2012) were amplified as a single fragment using PuReTaq™ Ready-To-Go polymerase chain reaction (PCR) beads (GE Biosciences Healthcare, NJ, USA), using a combination of ITS5/ITS1F/ITS1/LROR (forward) and ITS4/LR3 (reverse) primers (Vilgalys and Hester 1990; White et al. 1990; Gardes and Bruns 1993; Rehner and Samuels 1995), following established thermocycler parameters outlined previously (Promputtha and Miller 2010). The PCR products were purified to remove excess primers, dNTPs, and non-specific amplification products with the QIAquick PCR Purification Kit (QIAGEN Inc.). Purified PCR products were used in 11 µl sequencing reactions with BigDye® Terminators v3.1 (Applied Biosystems, Foster City, CA, USA) and sequenced bidirectionally using the above primer combinations. Sequences were generated on an Applied Biosystems 3730XL high-throughput capillary sequencer at the University of Illinois at Urbana-Champaign Biotech facility.

### Sequence alignment

Individual fragments were edited, and contigs for ITS and LSU were assembled using Sequencher 5.2.3 (Gene Codes Corp., Ann Arbor, MI, USA). Established guidelines (Nilsson et al. 2012) were followed for all newly generated ITS sequences.

### Designation of operational taxonomic units (OTUs)

For designation of operational taxonomic units (OTUs), the ITS sequences were subjected to a BLAST search against GenBank. A cut-off proxy of 98% was chosen for delineation of OTUs based on previous studies (Nilsson et al. 2006, 2008; Begerow et al. 2010; Gazis and Chaverri 2010; Koljalg et al. 2013). In addition, and where appropriate, knowledge of culture morphology was applied to the ITS data to make OTU designation more reliable. For designation of taxonomic names, the results of the ITS BLAST search using GenBank were interpreted with caution using modification of outlined criteria (Goncalves et al. 2012). For species identities, for ≥99–100%, genus and species were accepted; for 97% identity, genus and species were accepted by using the term (cf. = compares with); for ≤97–95%, only genus was accepted.

### Taxon sampling and phylogenetic analyses

The entire ITS region along with the 5.8S gene were sequenced together with the adjacent D1/D2 region of the 28S rRNA gene. ITS sequences were obtained for 41 representative isolates, whereas partial (D1/D2 region) of the 28S rRNA gene were obtained for 37 representative isolates. The ITS region was used to discriminate OTUs based on 98% sequence similarity and construction of species phylogeny, whereas the partial LSU region was used for phylogenetic analysis to determine the phylogenetic affinities of isolates with other closely related members of Ascomycota.

ITS sequences were obtained for 41 representative isolates and aligned using the multiple sequence alignment programme, MUSCLE® (Edgar 2004), with default parameters in operation. MUSCLE® was implemented using the program Seaview (Gouy et al. 2010). Prior to maximum likelihood (ML) analysis, ambiguous regions were removed from the final alignment using G blocks (Castresana 2000; Talavera and Castresana 2007). The ML analysis was performed using RAxML v. 7.0.4 (Stamatakis et al. 2008) on the CIPRES Portal (Miller et al. 2010.) v. 2.0 with the default rapid hill-climbing algorithm and GTR model employing 1000 fast bootstrap searches. Clades with bootstrap values ≥70% were considered as significant and of strong support (Hillis and Bull 1993).

Taxon sampling for ML analysis of the LSU dataset was obtained from previous study on the phylogenetic relationships of Ascomycota (Schoch et al. 2009). To visualize the higher taxonomic affiliations of the milk thistle fungal endophytes, partial LSU data (D1/D2 domains) from 37 representative isolates were incorporated into a core alignment of 189 taxa sampled from the Ascomycota (Schoch et al. 2009), which consisted of 1519 nucleotides. The final LSU alignment, after ambiguous characters were excluded, consisted of 1264 nucleotides. Representatives of four classes of Ascomycota (Dothideomycetes, Eurotiomycetes, Leotiomycetes, and Sordariomycetes) were included in the 226 taxa alignment, which included 37 representative isolates from the D1/D2 region of the LSU from milk thistle endophytes. Subsequently, ML analysis was conducted as outlined earlier using RAxML.

Combined ITS-LSU sequence data generated for this study were deposited in GenBank under following accession numbers (KM215615–KM215649), whereas complete ITS sequences were deposited in GenBank under accession numbers (KM215650–KM215653).

### General experimental procedures for chemical analyses

Nuclear magnetic resonance (NMR) experiments were conducted using an Agilent-700, JEOL ECA-500, and/or ECS-400 spectrometers (700, 500, or 400 MHz for ^1^H and 175, 125, or 100 MHz for ^13^C; Agilent Technologies, Santa Clara, CA, USA; JEOL Ltd., Tokyo, Japan). HRESIMS data were collected using an electrospray ionization source coupled to a Q-ToF Premier mass spectrometer (Waters Corp., Milford, MA, USA) or a LTQ Orbitrap XL system (Thermo Fisher Scientific, San Jose, CA, USA) in both positive and/or negative ionization modes by either direct injection or via a liquid chromatography/autosampler system that consisted of Acquity UPLC system (Waters Corp.). A CombiFlash Rf system using a RediSep Rf Si-gel Gold column (both from Teledyne-Isco, Lincoln, NE, USA) was employed for normal-phase flash column chromatography. High-performance liquid chromatography (HPLC) separations were performed using a Varian Prostar HPLC system (Varian Inc., Palo Alto, CA, USA) equipped with Prostar 210 pumps and a Prostar 335 photodiode array (PDA) detector using Galaxie Chromatography Workstation software (version 1.9.3.2, Varian Inc.). YMC ODS-A (Waters Corp.; 5 µm; columns of dimensions 250 × 20 mm, 250 × 10 mm, and 250 × 4.6 mm) or Kinetex C_18_ (Phenomenex, Torrance, CA, USA; 5 µm; columns of dimensions 250 × 21.2 mm and 250 × 4.6 mm) was used for preparative, semi-preparative, and analytical HPLC, respectively. For ultra-performance liquid chromatography (UPLC) analysis, a BEH C_18_ (Waters Corp.; 1.7 μm; 50 × 2.1 mm) column was used. Optical rotation data were acquired on a Rudolph Research Autopol III polarimeter. Electronic circular dichroism (ECD) data were collected using an Olis DSM 17CD spectrophotometer (Olis, Bogard, GA, USA). UV data were collected using a Varian Cary 100 Bio UV–vis spectrophotometer. The solvents were purchased from Fisher Scientific.

### Fungal cultures for solid-state fermentation

For chemical extractions, fungal cultures were grown on rice (VanderMolen et al. 2013). To make seed cultures for inoculating rice, a piece of fresh culture grown in MEA medium was excised from the leading edge of the colony and transferred to a liquid medium containing 2% soy peptone, 2% dextrose, and 1% yeast extract (YESD). Following incubation (7 days) at 22°C with agitation, the culture was used to inoculate 50 ml of rice media prepared using rice and twice the volume of rice with H_2_O in a 250 ml Erlenmeyer flask. This was incubated at 22°C until the cultures showed good growth (typically 14–21 days) to generate the screener cultures. For large-scale production of fungal cultures, four 250 ml Erlenmeyer flasks were inoculated using one seed culture for each flask.

### Chemical extraction of fungal cultures

To each 250 ml flask containing a fungal culture, 60 ml of 1:1 MeOH/CHCl_3_ were added. The cultures were chopped with a spatula and were shaken overnight (~16 h; RT) at ~100 rpm. The cultures were filtered by vacuum, and the remaining residues were washed with MeOH. To the filtrate, 90 ml of CHCl_3_ and 150 ml of water were added. The mixtures were stirred for 30 min and then transferred to separatory funnels. The bottom layers were drawn off into round bottom flasks, which were evaporated to dryness. These dried organic extracts were reconstituted with 100 ml of 1:1 MeOH/MeCN and 100 ml of hexanes and transferred to separatory funnels, where the biphasic solutions were shaken vigorously. The MeOH/MeCN layer was drawn off and evaporated to dryness under vacuum to obtain the organic extract. For scaled-up fermentation extracts (4 × 250 ml flasks), a protocol similar to that described above was employed, and the volumes of various solvents were adjusted accordingly.

### Isolation of secondary metabolites

Preliminary analysis of the crude extracts was performed using UPLC–PDA–high-resolution tandem mass spectrometric dereplication protocol, as detailed previously (El-Elimat et al. 2013). In an isolated case (G111), these data were sufficient for structural characterization. The following general protocol was used for chemical analyses of the remaining fungal extracts. Each crude extract was adsorbed on a minimum amount of Celite 545 (Acros Organics, Geel, Belgium) and dried before loading on to a cartridge. This adsorbed mixture was subjected to normal-phase silica gel flash column chromatography employing a step gradient with hexanes, CHCl_3_, and MeOH. Based on NMR and/or analytical HPLC profiles of the pooled column fractions, according to the UV and evaporative light scattering detector data, samples were then selected for further purification by preparative reversed-phase (RP) HPLC, resulting in the isolation of pure compounds. In a few cases (G323, G377, G410, G411, G412, and G413), the crude extract was directly subjected to preparative RP HPLC without a prior separation employing normal-phase column chromatography. Finally, the pure secondary metabolites (>95% purity by UPLC and/or NMR) were characterized using a suite of NMR and MS techniques. In cases where the complete NMR data for known compounds were not reported in the literature, they have been presented in the Supporting Information. In the process of structure elucidation, if the NMR data for selected compounds were recorded in deuterated solvents other than those reported in the literature, then those data have also been presented (Figure S1, Supporting Information). Since HPLC methods used were unique to each extract/fraction that was pursued for additional separation, the details have not been discussed here. However, the isolation protocols for the new compounds are presented below as representative examples.

Isolation of biscognin A (**1**), chlamydospordiol (**2**), and biscognin B (**3**)

A defatted organic extract of *Biscogniauxia mediterranea* (G410; 190 mg) was subjected to RP HPLC [gradient elution using MeCN:H_2_O (containing 0.1% HCOOH): 20–80% MeCN for 50 min and 80–100% for 20 min; λ = 210 and 254 nm] using a YMC ODS-A column (250 × 20 mm) eluting at a flow rate of 12 ml/min to afford chlamydospordiol (**2**; 16.38 mg, t_R_ 8.5 min), biscognin B (**3**; 2.86 mg, t_R_ 21.0 min), and biscognin A (**1**; 1.78 mg, t_R_ 22.5 min) in addition to other metabolites listed in Table 2.

Morphological characteristics and identification of *Biscogniauxia mediterranea* (G410)

Strain G410 was grown on PDA for 3 weeks. On PDA, the colony morphology resembled the description offered by Collado et al. (2001). Mycelium brown to reddish brown, mostly superficial to partly immersed; colony reverse pale brown to cinnamon. In our strain, the conidia were not produced abundantly. Based on the data obtained from ITS sequence, however, G410 was more closely related to *Biscogniauxia mediterranea* isolates sequenced by Sánchez-Ballesteros et al. (2000) than those generated by (Collado et al. 2001). This is interesting because ITS sequences from Sánchez’s study were generated from American collections of *B. mediterranea*, whereas those of Collado’s study were generated from European collections. It has been suggested that there is a high divergence between populations of *B. mediterranea* at both side of the Atlantic Ocean (Collado et al. 2001). The ITS sequences obtained from our strain G410 from the USA clustered with ITS sequences obtained from isolates of *B. mediterranea* in north, central, and south America (AF390413, AF390414, GQ377479, KF850388) as well as France (EF026134).

Isolation of monascuskaoliaone B (**4**) and monascuskaoliaone (**5**)

A defatted organic extract of *Microascus nidicola* (G377; 42 mg) was subjected to RP HPLC [gradient elution using MeCN:H_2_O (containing 0.1% HCOOH): 40–100% MeCN for 40 min; λ = 210 and 254 nm] using a YMC ODS-A column (250 × 20 mm) eluting at the same flow rate to afford monascuskaoliaone B (**5**; 0.87 mg, t_R_ 9.5 min) and monascuskaoliaone (**4**; 1.01 mg, t_R_ 25.5 min). A scaled-up fermentation extract of this isolate did not yield the above compounds, but instead, entirely different metabolites including *epi*-pestalamide A (**6**) were encountered as delineated in Table 2.

Morphological characteristics and identification of *Microascus nidicola* (G377)

Identification of strain G377 was based on the observation of cultural morphology as well as on micromorphological features. Based on its gross morphology, G377 showed a number of similarities to the genus *Microascus* Zukal (Barron et al. 1961) with close resemblance to *M. nidicola* Massee & E.S. Salmon (Abbott et al. 2002). These characters included black, globose to ovoid perithecia; peridial wall composed of *textura angularis* in surface view; evanescent asci; falcate to lunate ascospores (~5 × 2 µm); abundant perithecia produced on PDA media with orange to copper coloured ascospores produced in cirri at maturity (Barron et al. 1961; Malloch 1970; Abbott et al. 2002).

Biscognin A (**1**): white powder; [α]^25^_D_ ‒45 (*c* 0.11, CHCl_3_); UV/Vis (MeOH) λ_max_ (log *ε*) 214 (3.2), 286 (3.4) nm; ^1^H NMR data (CDCl_3_; 400 MHz) *δ* 5.43 (s, 3-H), 4.04 (dq, *J* = 8.3, 6.2, H-8), 3.80 (s, 4-OMe), 2.85 (dq, *J* = 8.3, 7.1, H-7), 1.90 (s, 5-Me), 1.25 (d, *J* = 7.1, H_3_-9), 1.17 (d, *J* = 6.2, 7-Me); ^13^C NMR (CDCl_3_; 100 MHz) *δ* 171.0 (C-4), 164.6 (C-2), 162.1 (C-6), 108.1 (C-5), 87.9 (C-3), 69.8 (C-8), 56.2 (4-OMe), 42.7 (C-7), 21.4 (C-9), 15.0 (7-Me), 9.3 (5-Me); see Figure S2; heteronuclear multiple bond correlations (HMBCs) (H-# → C-#): H-3 → C-2 (wk), C-4, and C-5; H-7 → 7-Me, C-8, and C-9; H-8 → C-6 (wk), C-7 (wk), and 7-Me; 4-OMe → C-4; 5-Me → C-4, C-5, and C-6; 7-Me → C-6, C-7, and C-8; H_3_-9 → C-7 and C-8; HRESIMS obsd *m/z* 213.1126 [M + H]^+^ (calcd for C_11_H_17_O_4_, 213.1121); preparation and ^1^H NMR data for Mosher’s esters of **1** are in Figure S5.

Biscognin B (**2**): white powder; UV/Vis (MeOH) λ_max_ (log *ε*) 222 (3.4), 277 (3.5), 326 (3.5) nm; ^1^H NMR data (CDCl_3_; 400 MHz) *δ* 5.52 (s, 3-H), 3.96 (s, 4-OMe), 2.33 (s, 7-Me), 2.07 (s, 8-Me); ^13^C NMR (CDCl_3_; 100 MHz) *δ* (C-#) 169.0 (C-4), 166.9 (C-8a), 162.7 (C-7), 160.6 (C-2 or C-5), 156.9 (C-2 or C-5), 106.1 (C-8), 97.3 (C-4a), 88.0 (C-3), 57.2 (4-OMe), 18.3 (7-Me), 9.8 (8-Me); see Figure S3; HMBC correlations (H-# → C-#) H-3 → C-2, C-4, C-4a, and C-5; 4-OMe→ C-4; 7-Me → C-7 and C-8; 8-Me → C-7, C-8, and C-8a; HRESIMS obsd *m/z* 223.0602 [M + H]^+^ (calcd for C_11_H_11_O_5_, 223.0601).

Monascuskaoliaone B (**4**): Colourless oil; [α]^25^_D_ +30 (*c* 0.067, MeOH); UV/Vis (MeOH) λ_max_ (log *ε*) 263 (3.4) nm; ECD (223 µM, MeOH) λ_max_ (Δ*ε*) 256 (+13) nm; ^1^H NMR data (CDCl_3_; 700 MHz); *δ* 5.46 (s, 4-H), 3.93 (t, *J* = 6.2, H_2_-7), 3.49 (m, H-15), 2.76 (m, H_2_-6), 1.69 (m, H_2_-8), 1.49 (m, H_α_-16), 1.41 (m, H_β_-16), 1.16‒1.48 m (m, H_2_-9 ‒ H_2_-14), 1.34 (s, 2-Me), 0.92 (t, *J* = 7.5, H_3_-17); ^13^C NMR (CDCl_3_; 175 MHz) *δ* 207.3 (C-3), 189.3 (C-5), 103.7 (C-4), 91.6 (C-2), 73.5 (C-15), 59.5 (C-7), 36.8 (C-8), 34.3 (C-6), 30.4 (C-16), 10.1 (C-17), Carbon NMR chemical shift for C-9‒C-14, could not be assigned with confidence but are listed here: *δ* 37.1, 29.56, 29.58, 29.4, 25.7, 23.1, 22.2; see Figure S4; Key HMBC correlations (H-# → C-#) 2- Me → C-2, C-3, and C-8; H-4 → C-2, C-3, and C-5; H_2_-6 → C-4, C-5, and C-7; H_2_-7 → C-5 and C-6; H_3_-17 → C-15 and C-16; HRESIMS obsd *m/z* 299.2213 [M + H]^+^ (calcd for C_17_H_31_O_4_, 299.2217); Preparation and ^1^H NMR data for Mosher’s esters of **4** are in Figure S5.

*Epi*-pestalamide A (**6**): Yellow oil; [α]^25^_D_ +15 (*c* 0.10, MeOH); ^1^H and ^13^C NMR data in (CD_3_)_2_CO were identical to those reported in literature (Ding et al. 2008).

### Bioassay

The effect of pure compounds on viability of human prostate carcinoma PC-3 cells was determined by the MTT [3-(4,5-dimethylthiazol-2-yl)-2, 5-diphenyl tetrazolium bromide] assay, and growth inhibition was assessed as the percent cell viability wherein vehicle-treated cells were taken as 100% viable. Human prostate carcinoma PC-3 cells were purchased from the American Type Culture Collection and cultured in RPMI 1640 medium, supplemented with 10% heat-inactivated foetal bovine serum and 100 U/ml penicillin G and 100 µg/ml streptomycin sulphate at 37°C in a humidified 5% CO_2_ incubator. Briefly, PC-3 cells (2500 cells per well) were seeded in a 96-well plate, allowed to grow overnight, and then treated with 25 µM of each test compounds for 48 h. Cells treated with dimethyl sulphoxide served as vehicle control. Statistical analysis was carried out with Sigma Stat software version 2.03 (Jandel scientific, San Rafael, CA, USA). One-way ANOVA followed by Tukey’s test was used for multiple comparisons, and a statistically significant difference was considered at *p* ≤ 0.05.

## Results

### Mycology

From 605 tissue samples, 41 isolates of endophytic fungi were recovered in culture. The total isolation frequency (the percent of tissue samples bearing cultivable endophytes) was 6.7%. The highest percentage of isolates was recovered from leaf tissue (16.5%) followed by root (3.6%), stems (1.9%), and seeds (1.4%). All of the fungal endophytes isolated from milk thistle belonged to the Ascomycota (Table 1; Figures S7 and S8).

**Table 1.** List of 25 identified OTUs, GenBank accession numbers, their origin, and abundance. In most cases, isolates were grouped based on 98% ITS rDNA sequence similarity and identified using BLAST search.

OTU identification	Species identification of most homologous sequence from GenBank based on BLAST search*	OTU number	Strain numbers/GenBank numbers	Origin/abundance			
				Stem	Leaf	Root	Seed
*Diaporthe* sp.	*Diaporthe cotoneastri* (NR 1197261)	(OTU 1)	G111 (KM215632)		1		1
*Thielavia* sp.	*Thielavia terricola* (AJ271579)	(OTU 2)	G323 (KM215635)		1		
*Penicillium* sp.	*Penicillium hispanicum* (JX841247)	(OTU 3)	G324 (KM215636)		1		
*Cladosporium* sp	*Cladosporium colombiae* (NR_119729)	(OTU 4)	G325 (KM215637)		1		
*Penicillium* sp.	*Penicillium copticola* (NR_1215161)	(OTU 5)	G339 (KM215638)		1		
*Myrothecium verrucaria*	*Myrothecium verrucaria* (CBS 328.52; AJ302003)	(OTU 6)	G340 (KM215639)		1		
*Chaetomidium arxii*	*Chaetomidium arxii* (JN709486)	(OTU 7)	G341 (KM215651)		1		
*Daldinia loculata*	*Daldinia loculata* (AF176959)	(OTU 8)	G343 (KM215641)		1		
*Zopfiella* sp.	*Zopfiella longicaudata* (GQ922541)	(OTU 9)	G367 (KM215644)		1		
*Microascus nidicola ^#^*	–	(OTU 10)	G377 (KM215645)		1		
*Trichophaea* sp.	*Trichophaea hybrida* (AF351582)	(OTU 11)	G78 (KM215630)/G379 (KM215647)	1	1		
*Biscogniauxia mediterranea*	*Biscogniauxia mediterranea* (AJ390413)	(OTU 12)	G410 (KM215652)		1		
*Biscogniauxia atropunctata*	*Biscogniauxia atropunctata* (AJ390411)	(OTU 13)	G411 (KM215648)		1		
*Penicillium* sp.	*Penicillium aculeatum* (AF033397)	(OTU 14)	G412 (KM215649)		1		
*Talaromyces minioluteus ^#^*	*Talaromyces minioluteus* (NR_121527.1)	(OTU 15)	G413 (KM215653)		1		
*Nemania serpens*	*Nemania serpens* (AJ390432)	(OTU 16)	G44 (KM215616)	1			
*Chaetomium* sp	*Chaetomium rectangulare* (HM365239)	(OTU 17)	G45 (KM215617)			1	
*Penicillium* sp.	*Penicillium restrictum* (NR_121239)	(OTU 18)	G342 (KM215640)			1	
*Alternaria* sp.	*Alternaria metachromatica* (AY762946)	(OTU 19)	G54 (KM215621)/G58 (KM215625)/G59 (KM215626)/G60 (KM5215627)/G62 (KM5215628)/G63 (KM5215629)		6		
*Alternaria* sp.	*Alternaria eichhorniae* (NR_111832)	(OTU 20)	G40 (KM215615)/G47 (KM215618)/G48 (KM215619)/G57 (KM215624)/G378 (KM215646)		4		1
*Alternaria* sp.	*Alternaria dauci* ((NR_077186)	(OTU 21)	G49 (KM215620)/G50 (KM215650)/G55 (KM215622)/G56 (KM215623)		4		
*Fusarium* sp.	*Fusarium fujikuroi* (NR_111889)	(OTU 22)	G344 (KM215642)/G345 (KM215643)		2		
*Penicillium restrictum ^#^*	*Penicillium restrictum* (NR_121239)	(OTU 23)	G85 (KF367458)	1			
*Acremonium* sp.	*Acremonium sclerotigenum* (FN706552)	(OTU 24)	G246 (KM215633)/G248 (KM215634)		2		
*Aspergillus iizukae ^#^*	*Aspergillus iizukae* (EF669597)	(OTU 25)	G77 (AB859956)/G82 (KM215631)	1	1		

Notes: *Fungal endophyte OTUs were tentatively assigned to either genus or species by matching the most homologous sequences in GenBank by BLAST search. Where possible, only authentic sequences (RefSeq) were used for assigning OTUs preferentially from type or other authentic cultures generated by taxonomic specialist published in high impact factor Mycology journals. When multiple species were found to have high sequence similarity or when <98% sequence homology was found with a published authentic sequence for which a culture was deposited in a public culture collection, we choose to take a more conservative approach and use only the genus name for OTU assignment. Furthermore, most OTU identifications were corroborated via their higher level phylogenetic placement using a portion of partial LSU sequence (D1/D2 region) (see Fig S8, Supporting Information). For OTU names followed by ‘#’ symbol, we used morphological characteristics of conidia or teleomorph formed in culture as well as sequence data from portions of partial LSU gene sequences (D1/D2) region and/or protein coding data in addition to ITS data in separately published studies by our research group to confirm OTU identification.

**Table 2.** Secondary metabolites isolated and/or identified from the selected endophytic fungal extracts of milk thistle.

Fungal identity based on ITS	Isolate number	Source (plant part)	Secondary metabolites	Characterization techniques (references)
*Alternaria* sp.	G40	Seed	9-O-methylalternariol, Pseurotin A, and Tyroscherin	NMR, UV, and MS (Hayakawa et al. 2004; Schmeda-Hirschmann et al. 2008; Tae et al. 2010; de Souza et al. 2013)
	G47	Leaf	Alternariol	NMR, UV, and MS (Koch et al. 2005)
	G48	Stem	Destruxin B, Homodestruxin B, and Antibiotic PF 1052	NMR, UV, and MS (Buchwaldt and Jensen 1991; Koyama et al. 2005)
	G55	Leaf	Euplectin, Coneuplectin, and Antibiotic PF 1052	NMR, UV, and MS (Ernst-Russell et al. 2000; Koyama et al. 2005)
*Alternaria* sp.	G49	Leaf	Antibiotic PF 1052	NMR, UV, and MS (Koyama et al. 2005)
	G50	Leaf	Destruxin B, Homodestruxin B, and Antibiotic PF 1052	NMR, UV, and MS (Buchwaldt and Jensen 1991; Koyama et al. 2005)
*Alternaria* sp.	G58	Leaf	Pyrenocines A and B, R-7-hydroxy-3-(S-1-hydroxyethyl)-5-methoxy-3,4-dimethylisobenzofuran-1(3H)-one, and R-4,8-dihydroxy-6-methoxy-4,5-dimethyl-3-methyleneisochroman-1-one	NMR, UV, and MS (Hashida et al. 2010; Tayone et al. 2011)
*Aspergillus iizukae* Sugiy	G77	Leaf	Methyl asterrate, Methyl 2,4-dichloroasterrate, Dihydrogeodin, Bisdechlorogeodin, Antibiotic SS 19508D, and Geodin	NMR, UV, and MS (Matsumoto et al. 1986; Tanaka et al. 1996; Hargreaves et al. 2002b; Sato et al. 2005; Lin et al. 2009)
*Diaporthe* sp.	G111	Seed	(-)-α-tetrahydro-bisabolen-2,5,6-triol	Dereplication: UV, retention time, and MS (El-Elimat et al. 2013)
*Thielavia* sp.	G323	Leaf	Thielavins B and C	NMR, UV, and MS (Kitahara et al. 1983; Jang et al. 2014)
*Penicillium* sp.	G324	Leaf	O-Methyldihydro gladiolic acid, 10,20-Dehydro[12,13-dehydroprolyl-2-(1,1-dimethylallyl)tryptophyl] diketopiperazine], 12,13-dehydroprolyl-2-(1,1-dimethylallyltryptophyl) diketopiperazine, Deoxybrevianamide E	NMR, UV, and MS (Steyn 1973; Ichihara et al. 1985)
*Penicillium* sp.	G339	Leaf	Bisorbicillinolide, Bisvertinolone, and Trichodimerol	NMR, UV, and MS (Trifonov et al. 1986; Andrade et al. 1992; Abe et al. 1998)
*Myrothecium verrucaria* (Alb. & Schwein.) Ditmar	G340	Leaf	Verrucarin A, Verrucarin J, Verrucarin L acetate, and Myrochromanol	NMR, UV, and MS (Tamm et al. 1972; Namikoshi et al. 2000; Liu et al. 2006)
*Chaetomidium arxii* Benny	G341	Leaf	Dihydro-5-(hydroxyphenylmethyl)-2(3H)-furanone	NMR, UV, and MS (Hargreaves et al. 2002a)
*Daldinia loculata* (Lév) Sacc.	G343		1,8-dimethoxynaphthalene	NMR, UV, and MS (Yang et al. 2008)
*Fusarium* sp.	G344 G345	Leaf Leaf	Beauvericin	NMR, UV, and MS (Zhan et al. 2007)
*Microascus nidicola* (Microascales)	G377	Leaf	Monascuskaoliaone, Nigragillin, Asperazine, Fonsecin, Fonsecin B, *Epi*-pestalamide A (6) Campyrone A, Campyrone C, Carbonarone A, and Monascuskaoliaone B (4)	NMR, UV, and MS (Caesar et al. 1969; Isogai et al. 1975; Varoglu et al. 1997; Zhang et al. 2007; Ding et al. 2008; Cheng et al. 2010; Shaaban et al. 2012; Mouafo Talontsi et al. 2013)
*Alternaria* sp.	G378	Leaf	Alternariol, 9-O-methylalternariol, and Altenuene	NMR, UV, and MS (Koch et al. 2005; Altemöller et al. 2006; de Souza et al. 2013)
*Biscogniauxia mediterranea* (De Not.) Kuntze	G410	Leaf	(3R)-5-methylmellein, (3R,4R)-*cis*-4-hydroxy-5-methylmellein, Acuminatopyrone, Chlamydospordiol, Chlamydosporol (major:minor mixture of diastereomers), and Cyclo-[L-Phe-L-Leu-L-Leu-L-Leu-L-Ile], Biscognin A (1) Biscognin B (3)	NMR, UV, optical rotation, and MS (Okuno et al. 1986; Solfrizzo et al. 1994; Visconti et al. 1994; Li et al. 2004)
*Biscogniauxia atropunctata* (Schwein.) Pouzar	G411	Leaf	(3R)-5-methylmellein, (3R)-5-formylmellein, (3R)-6-methoxy-5-methylmellein, and Cyclo-[L-Phe-L-Leu-L-Leu-L-Leu-L-Ile]	NMR, UV, and MS (Anderson et al. 1983; Okuno et al. 1986; Li et al. 2004; Sumarah et al. 2008)
*Penicillium* sp.	G412	Leaf	Paeciloxocin A, Tenellic acid C, Purpactin C, Purpactin C′, and Radiclonic acid	NMR, UV, and MS (Sassa et al. 1973; Seto et al. 1977; Nishida et al. 1991; Oh et al. 1999; Wen et al. 2010)

### ITS data

Based on 98% sequence similarity, 41 isolates were segregated into 25 OTUs (Figure S7; Table 1). The ITS alignment from representative isolates of fungal endophytes of milk thistle consisted of 870 nucleotides. The final ITS alignment, after the ambiguous regions were excluded, consisted of 436 nucleotides. The genera *Penicillium*, and *Biscogniauxia* were represented by more than one species.

### LSU phylogeny of fungal endophytes from milk thistle and their phylogenetic affinities with the Ascomycota

The LSU ML tree shows the phylogenetic diversity of milk thistle fungal endophytes and their affinities to the members of the Ascomycota (Figure S8). These endophytes were associated with four major classes of Ascomycota (Dothideomycetes, Eurotiomycetes, Leotiomycetes, and Sordariomycetes) (Figure S8). Within the Dothideomycetes, these endophytes (e.g., *Alternaria* sp. and *Cladosporium* sp.) showed phylogenetic affinities with the orders Pleosporales and Capnodiales. In the Eurotiomycetes, the milk thistle endophytes were nested within the Eurotiales and were represented by genera such as *Penicillium* and *Aspergillus.* The majority of the milk thistle endophyte isolates (35%) showed associations with the Sordariomycetes and were nested in diverse orders, including Diaporthales, Hypocreales, Microascales, Sordariales, and Xylariales. Only one isolate, *Trichophaea* sp., showed phylogenetic affinities with the Leotiales (Leotiomycetes) (Figure S8).

### Chemistry

**Figure 1.**
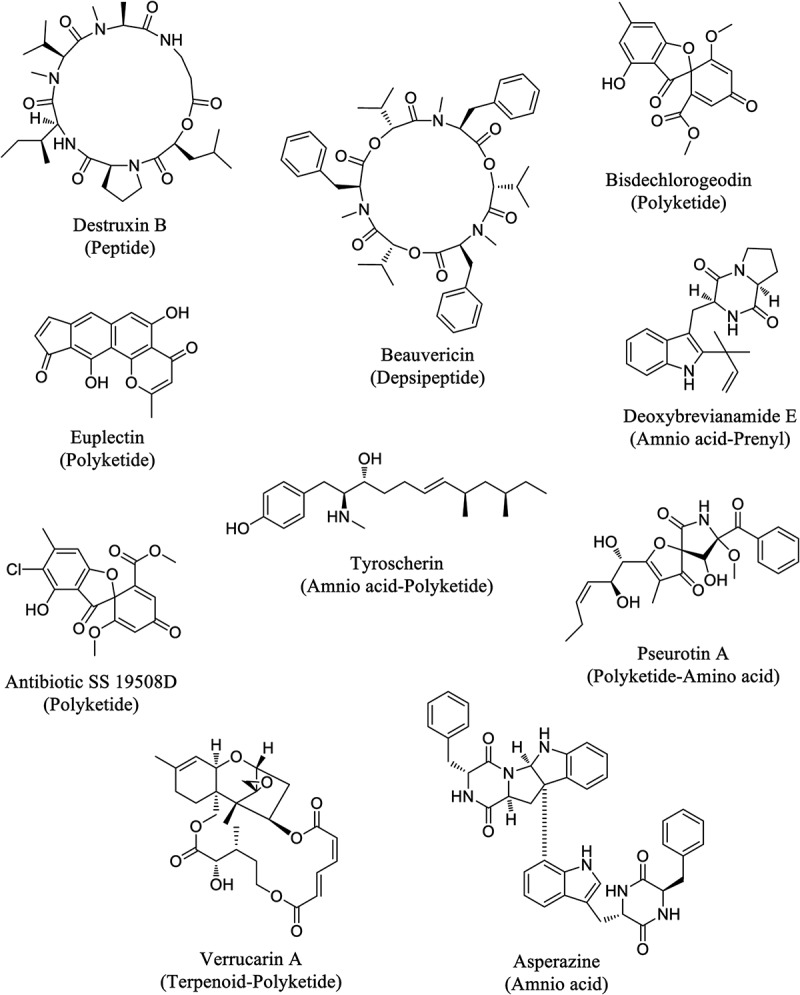
Structures of selected secondary metabolites identified from fungal endophytes of milk thistle and their biosynthetic classes.

Sixty-two secondary metabolites, representing a variety of structural classes, including polyketides, terpenoids, peptides, and those of mixed biosynthetic origins, were isolated and characterized from extracts of solid-phase cultures of endophytic fungi from milk thistle (Table 2 and Figure 1). Fifty-eight of these compounds were known in the literature and have been previously encountered from other fungal species (Figure S1). Many of these metabolites have been reported to possess diverse biological activities.

The flavonolignans from milk thistle have received considerable attention in recent years for their role in prostate cancer chemoprevention (Davis-Searles et al. 2005; Agarwal et al. 2006). In cases where the compounds from milk thistle fungal endophytes were isolated in sufficient purity (>95% by UPLC and/or NMR), they were tested *in vitro* for cytotoxic activity against the human prostate carcinoma (PC-3) cell line (Figure S9). PC-3 cells were treated with 25 µM of each compound for 48 h and analysed in the MTT assay.

Several compounds were found to exhibit moderate to strong cytotoxicity. Beauvericin, a cyclic hexadepsipeptide, was the most cytotoxic of all the evaluated metabolites (exhibiting 3% cell viability at 25 µM concentration) and has been reported in the literature to inhibit PC-3M cell proliferation/survival with an IC_50_ value of 3.8 µM (Zhan et al. 2007). Beauvericin has been shown to possess a variety of additional activities including entomopathogenic, antimicrobial, anticholesterolemic, and cytotoxic effects (Tomoda et al. 1992; Wang and Xu 2012). The antibiotic SS 19508D and a sample containing an inseparable major:minor (~10:1) mixture of euplectin and coneuplectin also showed significant cytotoxicity (~5% cell viability) in these assays. The biological activity of euplectin, originally isolated from *Flavoparmelia euplecta* (Ernst-Russell et al. 2000), has been evaluated against the murine P-815 mastocytoma cell line (IC_50_ 1.67 µg/ml), but neither euplectin nor antibiotic SS 19508D has been tested previously against models of prostate carcinoma. Other secondary metabolites, such as bisdechlorogeodin, verrucarin A, and tyroscherin, were found to be moderately cytotoxic (10–25% cell viability).

**Figure 2.**
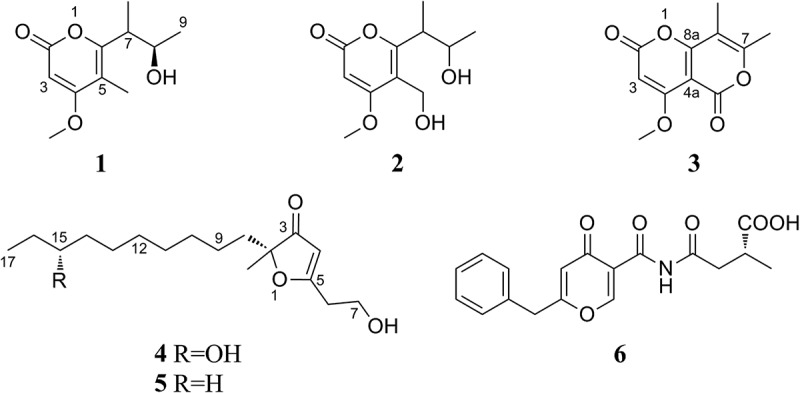
Structures of new secondary metabolites (1, 3, 4, and 6) and selected relevant analogues (2 and 5).

In addition to the known compounds, four new natural products [biscognin A (**1**), biscognin B (**3**), monascuskaoliaone B (**4**), and *epi*-pestalamide A (**6**)] were also obtained (Figure 2). Biscognin A (**1**) was assigned the molecular formula C_11_H_16_O_4_ on the basis of HRESIMS data, indicating an index of hydrogen deficiency of four. The ^1^H NMR spectrum showed signals for an olefinic proton, two methine protons (including an oxymethine signal), three methyl groups (a singlet and two doublets), and a methoxy group (Figure S2). All eleven carbons, including six *sp^3^*- and five *sp^2^*-hybridized carbons, were observed in the ^13^C NMR spectra. These signals closely resembled the data for a related compound, chlamydospordiol (**2**), which was also isolated as one of the major metabolites during investigation of the same endophytic fungus (G410). A set of mutually coupled diastereotopic protons (*δ* 4.52 and *δ* 4.29; *J *= 12.4 Hz), characteristic of an isolated oxymethylene unit in **2**, was replaced by a methyl singlet at *δ* 1.90 (5-Me) in compound **1**; this accounted for the only key difference in the ^1^H NMR spectra of the two compounds. A corresponding carbon signal (*δ* 9.3; 5-Me) was also observed in the ^13^C NMR spectrum of **1**. HMBC correlations from 5-Me to C-4 (*δ* 171.0), C-5 (*δ* 108.1), and C-6 (*δ* 162.1) confirmed the position of this methyl group, and the remaining HMBC correlations were consistent with the structure shown for **1**. The relative configuration at C-7 and C-8 could not be determined due to unsuccessful crystallization attempts limited by sample paucity as well as degradation over time. Mosher’s method (Hoye et al. 2007) was employed to independently assign the absolute configuration at C-8. However, esterification conditions resulted in the disappearance of the olefinic signal H-3 (*δ* 5.43) in the reaction product, suggesting that a modification of the ring system occurred during the reaction. Even so, the portion of the compound that contained the side chain appeared to remain intact, and the downfield chemical shift for H-8 (*δ* 5.17) in the reaction product compared with the *δ* 4.04 in **1** supported the formation of the acylated product. The measurable Δ*δ* values (Figure S5) observed for key signals of the *R*- and *S*-esters were consistent with the assignment of the *R*-configuration at C-9.

Biscognin B (**3**) had the molecular formula C_11_H_10_O_5_ (seven unsaturations), suggesting a structural motif similar to compounds **1** and **2** but with noticeable differences in the NMR data. Only four singlets, corresponding to an olefinic proton, a methoxy group, and two methyl groups, were observed in the ^1^H NMR spectrum of **3** (Figure S3). The ^13^C NMR spectrum showed signals for eleven carbons, eight of which correlated to *sp^2^*-hybridized carbons. The chemical shifts and HMBC correlations from H-3 to 4-OMe were indicative of a six-membered ring system identical to compounds **1** and **2**. Both methyl doublets (7-Me and 8-Me) showed HMBC correlations of the olefinic carbons C-7 (*δ* 162.7) and C-8 (*δ* 106.1). An additional HMBC correlation from 8-Me to C-8a (*δ* 166.9) confirmed the positions of the methyl groups on carbons adjacent to each other (C-7 and C-8) and established the connectivity of C-8 to C-8a. Only a -CO_2_ unit and two unsaturations were unaccounted. A four-bond HMBC correlations from H-3 to C-5 (*δ* 160.6 or *δ* 156.9) and chemical shift for C-4a (*δ* 97.3) as well as C-7 (*δ* 162.7) were consistent with a lactone linkage to C-4a via the carbonyl.

The molecular formula of monascuskaoliaone B (**4**) was determined to be C_17_H_30_O_4_ (three unsaturations) by HRESIMS. The ^1^H NMR spectrum exhibited signals for an olefinic proton, an isolated pair of mutually coupled methylene units, two methyl groups (singlet and triplet), and a cluster of aliphatic protons (Figure S4). A similar set of signals were also observed for a known natural product, monascuskaoliaone (**5**; molecular formula C_17_H_30_O_3_) that was also encountered during the course of our studies (Cheng et al. 2010). A comparison with the available literature data for **5** suggested an identical five-membered furan-derived ring system and a similar side chain at C-5 in the new metabolite **4**. HMBC correlations from olefinic proton H-4 (*δ* 5.46) to C-2 (*δ* 91.6), C-3 (*δ* 207.3), and C-5 (*δ* 189.3) as well as from aliphatic H_2_-6 (*δ* 2.76) to C-4 (*δ* 103.7), C-5, and C-7 (*δ* 59.8) supported the analogous assignment of this partial structure. HMBC correlations from the methyl group singlet (*δ* 1.34) to C-2, C-3, and C-8 (*δ* 36.8) established the location of this unit alpha to the carbonyl group. In comparison to **5**, key differences resided in chemical shifts for methylene protons of the decyl side chain and presence of an additional oxymethine signal (*δ* 3.49; H-15) in the ^1^H NMR spectrum of **4**. An HMBC correlation from H_3_-17 (*δ* 0.92) to carbon C-15 (*δ* 73.5), as well as the multiplicity of H_3_-17 (triplet), was consistent with the placement of a hydroxyl group at C-15.

The absolute configuration at C-15 was assigned as *R*- using the Mosher’s method (Hoye et al. 2007). Although both the primary and secondary alcohols were acylated on reaction with Mosher’s reagent, the distant location of the resulting ester groups was not expected to interfere with the assignment of absolute configuration at C-15. Due to extensive overlap in the ^1^H NMR signals for the aliphatic chain of **4**, the only useful Δ*δ*_S-R_ value (+0.12) for H_3_-17 was employed for the above assignment (Figure S5). Monascuskaoliaone (**5**) was isolated originally from *Monascus kaoliang* as a racemic mixture (Cheng et al. 2010). However, optically active samples for **4** and **5** were obtained from the extract of *Microascus nidicola* (G377). Since both compounds possessed an α, β-unsaturated ketone chromophore, a comparison of the calculated and experimental ECD spectra for both enantiomers was employed to determine the absolute configuration at C-2. Positive Cotton effects observed for both compounds were consistent with the assignment of *R*-configuration at C-2 (Figure S6). These results were also supported by comparisons between calculated and experimental-specific rotation values (Figure S6).

*Epi*-pestalamide A (**6**) had the molecular formula C_18_H_17_NO_6_ (eleven unsaturations). The ^1^H and ^13^C NMR data were identical to those reported for pestalamide A (Ding et al. 2008). However, comparison of the specific optical rotation values of **6** (+15) and pestalamide A (–12) suggested that **6** was an enantiomer of the reported compound.

## Discussion

This is the first comprehensive study of fungal endophytes from *Silybum marianum*, a plant of the Asteraceae. However, there have been few previous studies that have screened endophytes from Asteraceous plants (Romero et al. 2001; Shipunov et al. 2008; Gallo et al. 2009). Several genera of endophytes isolated in the present study (Table 1), such as *Aspergillus, Alternaria, Cladosporium, Diaporthe, Nemania*, and *Penicillium,* were also isolated from achenes of an invasive spotted knapweed, *Centaurea stoebe* (Shipunov et al. 2008). However, the species identities of the isolated fungi between *S. marianum* and *C. stoebe* were different. The genus *Alternaria*, isolated in this study, was also reported as an endophyte of *Smallanthus sonchifolius* (Asteraceae), which is a medicinal plant used for antidiabetic and antiinflammatory properties in Brazil, Japan, and the New Zealand (Gallo et al. 2009).

The majority of the fungal endophytes isolated in the present study can be classified as non-clavicipitaceous (class 3 endophytes) based on their host colonization patterns, mechanism of transmission, ecological functions, and *in planta* biodiversity (Rodriguez et al. 2009). Most of these class 3 non-clavicipitaceous endophytes belong to the Ascomycota (Pezizomycotina) (Figures S7 and S8). In our study, the isolated class 3 endophytes largely belonged to the Sordariomycetes and Dothideomycetes (Figure S8). Isolation of endophytes from these lineages of fungi agrees well with those reported in previous studies on fungal endophytes (Arnold 2007; Arnold and Lutzoni 2007; Hoffman and Arnold 2008; Gazis and Chaverri 2010; Linnakoski et al. 2012).

Despite screening 140 surface-sterilized milk thistle seeds, we only recovered 2 OTUs (*Alternaria* sp. and *Diaporthe* sp.) from the seeds. These fungi have been reported as endophytes previously from foliage of other plant species (Kurose et al. 2012; Douanla-Meli et al. 2013). Ganley and Newcombe (2006) found that only 2% of endophytes were recovered from 750 surface-sterilized seeds of *Pinus monticola.* Similarly, Arnold et al. (2003) were unsuccessful in obtaining endophytes from seeds of *Theobroma cacao*, while they found diverse endophytes associated with foliage of the same species. Conversely, Gallery et al. (2007) were able to recover ascomycetous endophytes from surface-sterilized seeds of a tropical tree (*Cecropia insignis*) using culture-independent methods, but failed to isolate fungi from seeds using culture-based methods. Thus, it is likely that seeds harbour a community of endophytes that may be more recalcitrant to culturing.

Similarly, we isolated only two OTUs (*Penicillium* sp. G342 and *Chaetomium* sp. G45) from surface-sterilized roots fragments. This may be due to the fact that only 55 root fragments were plated. Members of *Chaetomium* sp. are commonly found in the soil (Soytong et al. 2001), and therefore it was not surprising to find it associated with roots of milk thistle. *Chaetomium* spp. have also been reported as endophytes of many different plants such as soybean (de Souza Leite et al. 2013), wheat (Syed et al. 2009), and *Ginkgo biloba* (Qin et al. 2009). Various endophytic *Penicillium* spp. have been reported in the past to be associated with roots; for example, in *Picea mariana*, about 30 different *Penicillium* spp. have been isolated (Stone et al. 2000).

It is well known that endophytes may play a role in the growth and development of the host plant, in addition to providing protection against various sources, and are therefore potential sources of biologically active natural products (Strobel et al. 2004). Endophytic fungi inhabiting plants with a well-established ethnobotanical history represent a unique ecological group, since the medicinal effects of the plant could also be related to the resident endophytes (Kusari, Pandey, et al. 2013). In the last 10 years, there has been an upsurge of studies that have targeted fungal endophytes from medicinal plants for the isolation and characterization of novel metabolites (Wang et al. 2002; Tejesvi et al. 2006; Gomes-Figueiredo et al. 2007; Huang et al. 2008; Hyde and Soytong 2008; Naik et al. 2008; Krishnamurthy et al. 2009; Bills et al. 2012; Gond et al. 2012; Mishra et al. 2012; Vieira et al. 2012; Zubek et al. 2012; Miller, Qing, Sze, Neilan, et al. 2012; Aly et al. 2013; Chen et al. 2013; Ellsworth et al. 2013; Kusari, Kusari, et al. 2013). In the present study, we have isolated four new secondary metabolites [biscognin A (**1**), biscognin B (**3**), monascuskaoliaone B (**4**), and *epi*-pestalamide A (**6**)] from milk thistle endophytes, in addition to scores of known compounds that are members of a range of different classes of natural products. A plethora of biological activities for these compounds have been reported in the literature.

As part of our screening programme that involves testing the isolated metabolites in the available assays, and in view of well-documented chemopreventive properties of plant metabolites of milk thistle, selected pure compounds from fungal endophytes were also evaluated for cytotoxicity against a human prostate carcinoma cell line. The new compounds (**1**, **3**, and **6**) were found to be inactive in this assay, and compound **4** was not tested due to paucity of sample. It is likely that the new compounds isolated from *B. mediterranea*, in this study, could have a more natural function such as being an insecticidal agent or an insect signal molecule (pheromone). For example, a study by Pažoutová et al. (2013) showed that new compounds from *Daldinia hawksworthii* isolated from *Salix* spp. and a symbiont of woodwasp, *Xiphydria prolongata* exhibited weak cytotoxic and antimicrobial activities. The authors concluded that the compounds isolated from the Xylariaceous endophytes might have a more natural ecological function (Pažoutová et al. 2013). Thus, it would be interesting to test the bioactivity of biscognin A (**1**) and biscognin B (**3**) in the future as signalling molecules or insecticidal activity, since members of Xylariaceous endophytes from plants are linked to insects with respect to their life cycles (Bills et al. 2012). However, several compounds such as beauvericin, antibiotic SS 19508D, euplectin/coneuplectin (major:minor mixture), bisdechlorogeodin, verrucarin A, and tyroscherin exhibited moderate to strong cytotoxic activities (Figure S9). Most of these metabolites had not been examined previously in the PC-3 cell assay.

Several cytotoxic compounds that could contribute to the chemopreventive properties of milk thistle extracts were encountered during this study. However, an assessment of the therapeutic role of endophytes is debatable, partly due to the sporadic distribution of these microorganisms, both within the plant and based on the geographical location. Such variations have been reported for fungal endophytes inhabiting other plants (Collado et al. 1999; Taylor et al. 1999; Göre and Bucak 2007). Furthermore, extensive studies are required to link the role of the endophytic metabolites to the biological activities displayed by plant compounds as well as their role in plant physiology. Even so, the above results provide an in-depth analysis of the chemical mycology of the fungal endophytes from milk thistle. Our study extends beyond other such surveys, where typically only the crude extracts were tested for bioactivity (de Siqueira et al. 2011; Zhao et al. 2011; Carvalho et al. 2012). In the present investigation, scores of compounds were isolated, characterized, and tested in the cytotoxicity assay.

## Summary and conclusions

This is the first study that concurrently examined the chemistry and mycology of fungal endophytes from milk thistle. Our study, although based on a restricted geographical sampling, has demonstrated that fungal endophytes inhabit milk thistle leaves, stem, seed, and roots. Leaves harbour the most phylogenetically diverse fungal endophytes, belonging to four different classes in the Pezizomycotina, Ascomycota. Further sampling of milk thistle from other regions will likely yield more diverse fungal endophytes.

Of the four new and 58 known secondary metabolites encountered during the course of this study, about 10% of pure compounds exhibited moderate to strong toxicity against PC-3 cells. Chemical investigations aimed at structure elucidation of other new natural products isolated from milk thistle fungal endophytes in addition to those discussed here are underway.

## Disclosure statement

No potential conflict of interest was reported by the authors.

## Supplemental data

Supplemental data for this article can be accessed at 10.1080/21501203.2015.1009186.

## Supplementary Material

Supplementary_material.docx

## References

[CIT0001] Abbott SP, Lumley TC, Sigler L. 2002 Use of holomorph characters to delimit *Microascus nidicola* and *M. soppii* sp. nov., with notes on the genus *Pithoascus*. Mycologia. 94:362–369. doi:10.2307/3761813 21156506

[CIT0002] Abe N, Murata T, Hirota A 1998 Novel oxidized sorbicillin dimers with 1, 1-diphenyl-2-picrylhydrazyl-radical scavenging activity from a fungus. Biosci Biotechnol Biochem. 62:2120–2126. doi:10.1271/bbb.62.2120 27393585

[CIT0003] Agarwal R, Agarwal C, Ichikawa H, Singh RP, Aggarwal BB 2006 Anticancer potential of silymarin: from bench to bed side. Anticancer Res. 26:4457–4498. 17201169

[CIT0004] Altemöller M, Podlech J, Fenske D 2006 Total synthesis of altenuene and isoaltenuene. Eur J Org Chem. 2006:1678–1684. doi:10.1002/ejoc.200500904

[CIT0005] Aly AH, Debbab A, Proksch P 2011 Fungal endophytes: unique plant inhabitants with great promises. Appl Microbiol Biotechnol. 90:1829–1845. doi:10.1007/s00253-011-3270-y 21523479

[CIT0006] Aly AH, Debbab A, Proksch P 2013 Fungal endophytes - secret producers of bioactive plant metabolites. Pharmazie. 68:499–505. doi:10.1691/ph.2013.6517 23923629

[CIT0007] Aly AH, Edrada-Ebel R, Indriani ID, Wray V, Müller WEG, Totzke F, Zirrgiebel U, Schächtele C, Kubbutat MHG, Lin WH, et al. 2008 Cytotoxic metabolites from the fungal endophyte *Alternaria* sp. and their subsequent detection in its host plant *Polygonum senegalense*. J Nat Prod. 71:972–980. doi:10.1021/np070447m 18494522

[CIT0008] Anderson JR, Edwards RL, Whalley AJS 1983 Metabolites of the higher fungi. Part 21. 3-Methyl-3, 4-dihydroisocoumarins and related compounds from the ascomycete family Xylariaceae. J Chem Soc Perkin Trans. 1:2185–2192. doi:10.1039/P19830002185

[CIT0009] Andrade R, Ayer WA, Mebe PP 1992 The metabolites of *Trichoderma longibrachiatum*. Part 1. Isolation of the metabolites and the structure of trichodimerol. Can J Chem. 70:2526–2535. doi:10.1139/v92-320

[CIT0010] Arnold AE 2001 Fungal endophytes in neotropical trees: abundance, diversity, and ecological implications In: Ganeshaiah KN, Shaanker RU, Bawa KS, editors. Tropical ecosystems: structure, diversity and human welfare. New Delhi: Oxford & IBH; p. 739–743.

[CIT0011] Arnold AE 2007 Understanding the diversity of foliar endophytic fungi: progress, challenges, and frontiers. Fungal Biol Rev. 21:51–66. doi:10.1016/j.fbr.2007.05.003

[CIT0012] Arnold AE, Lutzoni F 2007 Diversity and host range of foliar fungal endophytes: are tropical leaves biodiversity hotspots? Ecology. 88:541–549. doi:10.1890/05-1459 17503580

[CIT0013] Arnold AE, Maynard Z, Gilbert GS 2001 Fungal endophytes in dicotyledonous neotropical trees: patterns of abundance and diversity. Mycol Res. 105:1502–1507. doi:10.1017/S0953756201004956

[CIT0014] Arnold AE, Maynard Z, Gilbert GS, Coley PD, Kursar TA 2000 Are tropical fungal endophytes hyperdiverse? Ecol Lett. 3:267–274. doi:10.1046/j.1461-0248.2000.00159.x

[CIT0015] Arnold AE, Mejía LC, Kyllo D, Rojas EI, Maynard Z, Robbins N, Herre EA 2003 Fungal endophytes limit pathogen damage in a tropical tree. Proc Natl Acad Sci USA. 100:15649–15654. doi:10.1073/pnas.2533483100 14671327 PMC307622

[CIT0016] Barron GL, Cain RF, Gilman JC 1961 The genus *Microascus*. Can J Bot. 39:1609–1631. doi:10.1139/b61-143

[CIT0017] Bascom-Slack CA, Arnold AE, Strobel SA 2012 Student-directed discovery of the plant microbiome and its products. Science. 338:485–486. doi:10.1126/Science.1215227 23112324

[CIT0018] Begerow D, Nilsson H, Unterseher M, Maier W 2010 Current state and perspectives of fungal DNA barcoding and rapid identification procedures. Appl Microbiol Biotechnol. 87:99–108. doi:10.1007/s00253-010-2585-4 20405123

[CIT0019] Bills GF, González-Menéndez V, Martín J, Platas G, Fournier J, Peršoh D, Stadler M, Figueras M-J 2012 *Hypoxylon pulicicidum* sp. nov. (Ascomycota, Xylariales), a pantropical insecticide-producing endophyte. PLoS ONE. 7:e46687. doi:10.1371/journal.pone.0046687 23056404 PMC3467290

[CIT0020] Blaising J, Levy PL, Gondeau C, Phelip C, Varbanov M, Teissier E, Ruggiero F, Polyak SJ, Oberlies NH, Ivanovic T, et al. 2013 Silibinin inhibits hepatitis C virus entry into hepatocytes by hindering clathrin-dependent trafficking. Cell Microbiol. 15:1866–1882. doi:10.1111/Cmi.12155 23701235

[CIT0021] Blumenthal M, Cavaliere C, Ferrier GKL 2006 Total sales of herbal supplements in United States show steady growth. HerbalGram. 71:64–66.

[CIT0022] Brantley SJ, Graf TN, Oberlies NH, Paine MF 2013 A systematic approach to evaluate herb-drug interaction mechanisms: investigation of milk thistle extracts and eight isolated constituents as CYP3A inhibitors. Drug Metab Dispos. 41:1662–1670. doi:10.1124/Dmd.113.052563 23801821 PMC3876807

[CIT0023] Brantley SJ, Gufford BT, Dua R, Fediuk DJ, Graf TN, Scarlett YV, Frederick KS, Fisher MB, Oberlies NH, Paine MF 2014 Physiologically based pharmacokinetic modeling framework for quantitative prediction of an herb-drug interaction. CPT Pharmacometrics Syst Pharmacol. 3:e107. doi:10.1038/psp.2013.69 24670388 PMC4042458

[CIT0024] Brantley SJ, Oberlies NH, Kroll DJ, Paine MF 2010 Two flavonolignans from milk thistle (*Silybum marianum*) inhibit CYP2C9-mediated warfarin metabolism at clinically achievable concentrations. J Pharmacol Exp Ther. 332:1081–1087. doi:10.1124/Jpet.109.161927 19934397 PMC2835426

[CIT0025] Buchwaldt L, Jensen JS 1991 HPLC purification of destruxins produced by *Alternaria brassicae* in culture and leaves of *Brassica napus*. Phytochemistry. 30:2311–2316. doi:10.1016/0031-9422(91)83638-2

[CIT0026] Caesar F, Jansson K, Mutschler E 1969 Nigragillin, a new alkaloid from the *Aspergillus niger* group. 1. Isolation and structure clarification of nigragillin and a dioxopiperazine. Pharm Acta Helv. 44:676–690. 5364772

[CIT0027] Carroll GC 1991 Beyond pest deterrence. Alternative strategies and hidden costs of endophytic mutualisms in vascular plants In: Andrews JH, Hirano SS, editors. Microbial ecology of leaves. New York (NY): Springer; p. 358–375. doi:10.1007/978-1-4612-3168-4_18

[CIT0028] Carvalho CR, Gonçalves VN, Pereira CB, Johann S, Galliza IV, Alves TMA, Rabello A, Sobral MEG, Zani CL, Rosa CA, et al. 2012 The diversity, antimicrobial and anticancer activity of endophytic fungi associated with the medicinal plant *Stryphnodendron adstringens* (Mart.) Coville (Fabaceae) from the Brazilian savannah. Symbiosis. 57:95–107. doi:10.1007/s13199-012-0182-2

[CIT0029] Castresana J 2000 Selection of conserved blocks from multiple alignments for their use in phylogenetic analysis. Mol Biol Evol. 17:540–552. doi:10.1093/oxfordjournals.molbev.a026334 10742046

[CIT0030] Chen J, Hu K-X, Hou X-Q, Guo S-X 2011 Endophytic fungi assemblages from 10 *Dendrobium* medicinal plants (Orchidaceae). World J Microbiol Biotechnol. 27:1009–1016. doi:10.1007/s11274-010-0544-y

[CIT0031] Chen J, Zhang L-C, Xing Y-M, Wang Y-Q, Xing X-K, Zhang D-W, Liang H-Q, Guo S-X, Lespinet O 2013 Diversity and taxonomy of endophytic xylariaceous fungi from medicinal plants of *Dendrobium* (Orchidaceae). PLoS ONE. 8:e58268. doi:10.1371/journal.pone.0058268 23472167 PMC3589337

[CIT0032] Cheng M-J, Wu M-D, Yang P-S, Chen-J-J, Chen I-S, Chen Y-L, Yuan G-F 2010 Secondary metabolites isolated from the fungus *Monascus kaoliang*-fermented rice. J Chil Chem Soc. 55:107–110. doi:10.4067/S0717-97072010000100025

[CIT0033] Chomcheon P, Sriubolmas N, Wiyakrutta S, Ngamrojanavanich N, Chaichit N, Mahidol C, Ruchirawat S, Kittakoop P 2006 Cyclopentenones, scaffolds for organic syntheses produced by the endophytic fungus mitosporic dothideomycete sp. LRUB20. J Nat Prod. 69:1351–1353. doi:10.1021/np060148h 16989533

[CIT0034] Chomcheon P, Wiyakrutta S, Sriubolmas N, Ngamrojanavanich N, Isarangkul D, Kittakoop P 2005 3-Nitropropionic acid (3-NPA), a potent antimycobacterial agent from endophytic fungi: is 3-NPA in some plants produced by endophytes? J Nat Prod. 68:1103–1105. doi:10.1021/np050036a 16038559

[CIT0035] Collado J, Platas G, González I, Peláez F 1999 Geographical and seasonal influences on the distribution of fungal endophytes in *Quercus ilex*. New Phytol. 144:525–532. doi:10.1046/j.1469-8137.1999.00533.x 33862861

[CIT0036] Collado J, Platas G, Peláez F 2001 Identification of an endophytic *Nodulisporium* sp. from *Quercus ilex* in central Spain as the anamorph of *Biscogniauxia mediterranea* by rDNA sequence analysis and effect of different ecological factors on distribution of the fungus. Mycologia. 93:875–886. doi:10.2307/3761753

[CIT0037] Davis-Searles PR, Nakanishi Y, Kim N-C, Graf TN, Oberlies NH, Wani MC, Wall ME, Agarwal R, Kroll DJ 2005 Milk thistle and prostate cancer: differential effects of pure flavonolignans from *Silybum marianum* on antiproliferative end points in human prostate carcinoma cells. Cancer Res. 65:4448–4457. doi:10.1158/0008-5472.CAN-04-4662 15899838

[CIT0038] de Siqueira VM, Conti R, De Araújo JM, Souza-Motta CM 2011 Endophytic fungi from the medicinal plant *Lippia sidoides* Cham. and their antimicrobial activity. Symbiosis. 53:89–95. doi:10.1007/s13199-011-0113-7

[CIT0039] de Souza GD, Mithöfer A, Daolio C, Schneider B, Rodrigues-Filho E 2013 Identification of *Alternaria alternata* mycotoxins by LC-SPE-NMR and their cytotoxic effects to soybean (*Glycine max*) cell suspension culture. Molecules. 18:2528–2538. doi:10.3390/molecules18032528 23442929 PMC6270395

[CIT0040] de Souza Leite T, Cnossen-Fassoni A, Pereira OL, Mizubuti ESG, De Araújo EF, De Queiroz MV 2013 Novel and highly diverse fungal endophytes in soybean revealed by the consortium of two different techniques. J Microbiol. 51:56–69. doi:10.1007/s12275-013-2356-x 23456713

[CIT0041] Debbab A, Aly AH, Edrada-Ebel R, Wray V, Müller WEG, Totzke F, Zirrgiebel U, Schächtele C, Kubbutat MHG, Lin WH, et al. 2009 Bioactive metabolites from the endophytic fungus *Stemphylium globuliferum* isolated from *Mentha pulegium*. J Nat Prod. 72:626–631. doi:10.1021/np8004997 19271717

[CIT0042] Deep G, Gangar SC, Oberlies NH, Kroll DJ, Agarwal R 2010 Isosilybin A induces apoptosis in human prostate cancer cells via targeting Akt, NF-κB, and androgen receptor signaling. Mol Carcinogen. 49:902–912. doi:10.1002/Mc.20670 PMC3924888 20721970

[CIT0043] Deep G, Oberlies NH, Kroll DJ, Agarwal R 2007 Isosilybin B and isosilybin A inhibit growth, induce G1 arrest and cause apoptosis in human prostate cancer LNCaP and 22Rv1 cells. Carcinogenesis. 28:1533–1542. doi:10.1093/carcin/bgm069 17389612

[CIT0044] Deep G, Oberlies NH, Kroll DJ, Agarwal R 2008 Isosilybin B causes androgen receptor degradation in human prostate carcinoma cells via PI3K-Akt-Mdm2-mediated pathway. Oncogene. 27:3986–3998. doi:10.1038/onc.2008.45 18332867

[CIT0045] Deep G, Raina K, Singh RP, Oberlies NH, Kroll DJ, Agarwal R 2008 Isosilibinin inhibits advanced human prostate cancer growth in athymic nude mice: comparison with silymarin and silibinin. Int J Cancer. 123:2750–2758. doi:10.1002/Ijc.23879 18798272

[CIT0046] Ding G, Jiang L, Guo L, Chen X, Zhang H, Che Y 2008 Pestalazines and pestalamides, bioactive metabolites from the plant pathogenic fungus *Pestalotiopsis theae*. J Nat Prod. 71:1861–1865. doi:10.1021/np800357g 18855443

[CIT0047] Douanla-Meli C, Langer E, Talontsi Mouafo F 2013 Fungal endophyte diversity and community patterns in healthy and yellowing leaves of *Citrus limon*. Fungal Ecol. 6:212–222. doi:10.1016/j.funeco.2013.01.004

[CIT0048] Edgar RC 2004 MUSCLE: multiple sequence alignment with high accuracy and high throughput. Nucleic Acids Res. 32:1792–1797. doi:10.1093/Nar/Gkh340 15034147 PMC390337

[CIT0049] El-Elimat T, Figueroa M, Ehrmann BM, Cech NB, Pearce CJ, Oberlies NH 2013 High-resolution MS, MS/MS, and UV database of fungal secondary metabolites as a dereplication protocol for bioactive natural products. J Nat Prod. 76:1709–1716. doi:10.1021/np4004307 23947912 PMC3856222

[CIT0050] El-Elimat T, Raja HA, Graf TN, Faeth SH, Cech NB, Oberlies NH 2014 Flavonolignans from *Aspergillus iizukae*, a fungal endophyte of milk thistle (*Silybum marianum*). J Nat Prod. 77:193–199. doi:10.1021/np400955q 24456525

[CIT0051] Ellsworth KT, Clark TN, Gray CA, Johnson JA 2013 Isolation and bioassay screening of medicinal plant endophytes from eastern Canada. Can J Microbiol. 59:761–765. doi:10.1139/cjm-2013-0639 24206359

[CIT0052] Ernst-Russell MA, Chai CLL, Wardlaw JH, Elix JA 2000 Euplectin and Coneuplectin, new naphthopyrones from the Llchen *Flavoparmelia euplecta*. J Nat Prod. 63:129–131. doi:10.1021/np9903245 10650094

[CIT0053] Faeth SH, Hammon KE 1997 Fungal endophytes in oak trees: long-term patterns of abundance and associations with leafminers. Ecology. 78:810–819. doi:10.1890/0012-9658(1997)078[0810:FEIOTL]2.0.CO;2

[CIT0054] Figueroa M, Jarmusch AK, Raja HA, El-Elimat T, Kavanaugh JS, Horswill AR, Cooks RG, Cech NB, Oberlies NH 2014 Polyhydroxyanthraquinones as quorum sensing inhibitors from the guttates of *Penicillium restrictum* and their analysis by desorption electrospray ionization mass spectrometry. J Nat Prod. 77:1351–1358. doi:10.1021/np5000704 24911880 PMC4073659

[CIT0055] Gallery RE, Dalling JW, Arnold AE 2007 Diversity, host affinity, and distribution of seed-infecting fungi: a case study with *Cecropia*. Ecology. 88:582–588. doi:10.1890/05-1207 17503585

[CIT0056] Gallo MBC, Chagas FO, Almeida MO, Macedo CC, Cavalcanti BC, Barros WA, De Moraes MO, Costa-Lotufo LV, Pessoa C, Bastos JK, et al. 2009 Endophytic fungi found in association with *Smallanthus sonchifolius* (Asteraceae) as resourceful producers of cytotoxic bioactive natural products. J Basic Microbiol. 49:142–151. doi:10.1002/jobm.200800093 18798172

[CIT0057] Ganley RJ, Newcombe G 2006 Fungal endophytes in seeds and needles of *Pinus monticola*. Mycol Res. 110:318–327. doi:10.1016/j.mycres.2005.10.005 16492396

[CIT0058] Gardes M, Bruns TD 1993 ITS primers with enhanced specificity for basidiomycetes - application to the identification of mycorrhizae and rusts. Mol Ecol. 2:113–118. doi:10.1111/j.1365-294X.1993.tb00005.x 8180733

[CIT0059] Gazis R, Chaverri P 2010 Diversity of fungal endophytes in leaves and stems of wild rubber trees (*Hevea brasiliensis*) in Peru. Fungal Ecol. 3:240–254. doi:10.1016/j.funeco.2009.12.001

[CIT0060] Gazis R, Miadlikowska J, Lutzoni F, Arnold AE, Chaverri P 2012 Culture-based study of endophytes associated with rubber trees in Peru reveals a new class of Pezizomycotina: Xylonomycetes. Mol Phylogenet Evol. 65:294–304. doi:10.1016/J.Ympev.2012.06.019 22772026

[CIT0061] Gomes-Figueiredo J, Pimentel IC, Vicente VA, Pie MR, Kava-Cordeiro V, Galli-Terasawa L, Pereira JO, de Souza AQL, Glienke C 2007 Bioprospecting highly diverse endophytic *Pestalotiopsis* spp. with antibacterial properties from *Maytenus ilicifolia*, a medicinal plant from Brazil. Can J Microbiol. 53:1123–1132. doi:10.1139/W07-078 18026204

[CIT0062] Goncalves VN, Vaz ABM, Rosa CA, Rosa LH 2012 Diversity and distribution of fungal communities in lakes of Antarctica. FEMS Microbiol Ecol. 82:459–471. doi:10.1111/J.1574-6941.2012.01424.X 22671312

[CIT0063] Gond SK, Mishra A, Sharma VK, Verma SK, Kumar J, Kharwar RN, Kumar A 2012 Diversity and antimicrobial activity of endophytic fungi isolated from *Nyctanthes arbor-tristis*, a well-known medicinal plant of India. Mycoscience. 53:113–121. doi:10.1007/s10267-011-0146-z

[CIT0064] Göre ME, Bucak C 2007 Geographical and seasonal influences on the distribution of fungal endophytes in *Laurus nobilis*. Forest Pathol. 37:281–288. doi:10.1111/j.1439-0329.2007.00509.x

[CIT0065] Gouy M, Guindon S, Gascuel O 2010 SeaView version 4: a multiplatform graphical user interface for sequence alignment and phylogenetic tree building. Mol Biol Evol. 27:221–224. doi:10.1093/Molbev/Msp259 19854763

[CIT0066] Graf TN, Wani MC, Agarwal R, Kroll D, Oberlies NH 2007 Gram-scale purification of flavonolignan diastereoisomers from *Silybum marianum* (milk thistle) extract in support of preclinical in vivo studies for prostate cancer chemoprevention. Planta Med. 73:1495–1501. doi:10.1055/S-2007-990239 17948171

[CIT0067] Gu W, Ge HM, Song YC, Ding H, Zhu HL, Zhao XA, Tan RX 2007 Cytotoxic benzo[*j*]fluoranthene metabolites from *Hypoxylon truncatum* IFB-18, an endophyte of *Artemisia annua*. J Nat Prod. 70:114–117. doi:10.1021/np0604127 17253861

[CIT0068] Hargreaves J, Park J, Ghisalberti EL, Sivasithamparam K, Skelton BW, White AH 2002a Bioactive butyrolactones from fungi. Aust J Chem. 55:625–627. doi:10.1071/CH02154

[CIT0069] Hargreaves J, Park J-O, Ghisalberti EL, Sivasithamparam K, Skelton BW, White AH 2002b New chlorinated diphenyl ethers from an *Aspergillus* species. J Nat Prod. 65:7–10. doi:10.1021/np0102758 11809055

[CIT0070] Hashida J, Niitsuma M, Iwatsuki M, Mori M, Ishiyama A, Namatame M, Nishihara-Tsukashima A, Nonaka K, Ui H, Masuma R, et al. 2010 Pyrenocine I, a new pyrenocine analog produced by *Paecilomyces* sp. FKI-3573. J Antibiot. 63:559–561. doi:10.1038/ja.2010.65 20588297

[CIT0071] Hayakawa Y, Yamashita T, Mori T, Nagai K, Shin-Ya K, Watanabe H 2004 Structure of tyroscherin, an antitumor antibiotic against IGF-1-dependent cells from Pseudallescheria sp. J Antibiot. 57:634–638. doi:10.7164/antibiotics.57.634 15638323

[CIT0072] Hillis DM, Bull JJ 1993 An empirical-test of bootstrapping as a method for assessing confidence in phylogenetic analysis. Syst Biol. 42:182–192. doi:10.1093/sysbio/42.2.182

[CIT0073] Hoffman MT, Arnold AE 2008 Geographic locality and host identity shape fungal endophyte communities in cupressaceous trees. Mycol Res. 112:331–344. doi:10.1016/J.Mycres.2007.10.014 18308531

[CIT0074] Hoye TR, Jeffrey CS, Shao F 2007 Mosher ester analysis for the determination of absolute configuration of stereogenic (chiral) carbinol carbons. Nat Protoc. 2:2451–2458. doi:10.1038/nprot.2007.354. 17947986

[CIT0075] Huang WY, Cai YZ, Hyde KD, Corke H, Sun M 2008 Biodiversity of endophytic fungi associated with 29 traditional Chinese medicinal plants. Fung Divers. 33:61–75.

[CIT0076] Hyde KD, Soytong K 2008 The fungal endophyte dilemma. Fung Divers. 33:163–173.

[CIT0077] Ichihara A, Sawamura S, Kawakami Y, Sakamura S 1985 Dihydrogladiolic acid, another phytotoxin from *Phoma asparagi* Sacc. Agric Biol Chem. 49:1891–1892. doi:10.1271/bbb1961.49.1891

[CIT0078] Isogai A, Horii T, Suzuki A, Murakoshi S, Ikeda K, Sato S, Tamura S 1975 Isolation and identification of Nigragillin as a insecticidal metabolite produced by a *Aspergillus niger*. Agric Biol Chem. 39:739–740. doi:10.1271/bbb1961.39.739

[CIT0079] Jang J-P, Jang J-H, Oh M, Son S, Kim SM, Kim H-M, Shin K-S, Oh H, Soung NK, Hong Y-S, et al. 2014 Inhibition of indoleamine 2,3-dioxygenase by thielavin derivatives from a soil fungus, *Coniochaeta* sp 10F058. J Antibiot. 67:331–333. doi:10.1038/Ja.2013.134 24326340

[CIT0080] Kharwar RN, Mishra A, Gond SK, Stierle A, Stierle D 2011 Anticancer compounds derived from fungal endophytes: their importance and future challenges. Nat Prod Rep. 28:1208–1228. doi:10.1039/C1np00008j 21455524

[CIT0081] Kim N-C, Graf TN, Sparacino CM, Wani MC, Wall ME 2003 Complete isolation and characterization of silybins and isosilybins from milk thistle (*Silybum marianum*). Org Biomol Chem. 1:1684–1689. doi:10.1039/B300099k 12926355

[CIT0082] Kitahara N, Haruyama H, Hata T, Takahashi S 1983 The structures of thielavins A, B and C. Prostaglandin synthetase inhibitors from fungi. J Antibiot. 36:599–600. doi:10.7164/antibiotics.36.599 6409872

[CIT0083] Koch K, Podlech J, Pfeiffer E, Metzler M 2005 Total synthesis of alternariol. J Org Chem. 70:3275–3276. doi:10.1021/jo050075r 15822993

[CIT0084] Koljalg U, Nilsson RH, Abarenkov K, Tedersoo L, Taylor AFS, Bahram M, Bates ST, Bruns TD, Bengtsson-Palme J, Callaghan TM, et al. 2013 Towards a unified paradigm for sequence-based identification of fungi. Mol Ecol. 22:5271–5277. doi:10.1111/Mec.12481 24112409

[CIT0085] Koyama N, Nagahiro T, Yamaguchi Y, Ohshiro T, Masuma R, Tomoda H, Omura S 2005 Spylidone, a novel inhibitor of lipid droplet accumulation in mouse macrophages produced by *Phoma* sp. FKI-1840. J Antibiot. 58:338–345. doi:10.1038/ja.2005.42 16060386

[CIT0086] Krishnamurthy YL, Shashikala J, Shankar Naik B 2009 Diversity and seasonal variation of endophytic fungal communities associated with some medicinal trees of the Western Ghats, Southern India. Sydowia. 61:255–266.

[CIT0087] Kroll DJ, Shaw HS, Oberlies NH 2007 Milk thistle nomenclature: why it matters in cancer research and pharmacokinetic studies. Integr Cancer Ther. 6:110–119. doi:10.1177/1534735407301825 17548790

[CIT0088] Kumar DSS, Hyde KD 2004 Biodiversity and tissue-recurrence of endophytic fungi in *Tripterygium wilfordii*. Fung Divers. 17:69–90.

[CIT0089] Kurose D, Furuya N, Tsuchiya K, Tsushima S, Evans H 2012 Endophytic fungi associated with *Fallopia japonica* (Polygonaceae) in Japan and their interactions with *Puccinia polygoni-amphibii* var. *tovariae*, a candidate for classical biological control. Fungal Biol. 116:785–791. doi:10.1016/j.funbio.2012.04.011 22749165

[CIT0090] Kusari P, Kusari S, Spiteller M, Kayser O 2013 Endophytic fungi harbored in *Cannabis sativa* L.: diversity and potential as biocontrol agents against host plant-specific phytopathogens. Fung Divers. 60:137–151. doi:10.1007/s13225-012-0216-3

[CIT0091] Kusari S, Pandey SP, Spiteller M 2013 Untapped mutualistic paradigms linking host plant and endophytic fungal production of similar bioactive secondary metabolites. Phytochemistry. 91:81–87. doi:10.1016/j.phytochem.2012.07.021 22954732

[CIT0092] Kusari S, Zühlke S, Spiteller M 2009 An endophytic fungus from *Camptotheca acuminata* that produces camptothecin and analogues. J Nat Prod. 72:2–7. doi:10.1021/np800455b 19119919

[CIT0093] Langenfeld A, Prado S, Nay B, Cruaud C, Lacoste S, Bury E, Hachette F, Hosoya T, Dupont J 2013 Geographic locality greatly influences fungal endophyte communities in *Cephalotaxus harringtonia*. Fungal Biol. 117:124–136. doi:10.1016/J.Funbio.2012.12.005. 23452950

[CIT0094] Lee DYW, Liu L 2003 Molecular structure and stereochemistry of silybin A, silybin B, isosilybin A, and isosilybin B, isolated from *Silybum marianum* (milk thistle). J Nat Prod. 66:1171–1174. doi:10.1021/Np030163b 14510591

[CIT0095] Li H-J, Lin Y-C, Yao J-H, Vrijmoed L, Jones EBG 2004 Two new metabolites from the mangrove endophytic fungus no. 2524. J Asian Nat Prod Res. 6:185–191. doi:10.1080/102860201653237 15224415

[CIT0096] Lin T, Lu CH, Shen YM 2009 Secondary metabolites of *Aspergillus* sp F1, a commensal fungal strain of *Trewia nudiflora*. Nat Prod Res. 23:77–85. doi:10.1080/14786410701852826 19140073

[CIT0097] Linnakoski R, Puhakka-Tarvainen H, Pappinen A 2012 Endophytic fungi isolated from *Khaya anthotheca* in Ghana. Fungal Ecol. 5:298–308. doi:10.1016/j.funeco.2011.08.006

[CIT0098] Liu J, Huang L, Ye Y, Zou W, Guo Z, Tan R 2006 Antifungal and new metabolites of *Myrothecium* sp. Z16, a fungus associated with white croaker *Argyrosomus argentatus*. J Appl Microbiol. 100:195–202. doi:10.1111/j.1365-2672.2005.02760.x 16405700

[CIT0099] Liu K-L, Porras-Alfaro A, Kuske CR, Eichorst SA, Xie G 2012 Accurate, rapid taxonomic classification of fungal large-subunit rRNA genes. Appl Environ Microbiol. 78:1523–1533. doi:10.1128/AEM.06826-11 22194300 PMC3294464

[CIT0100] Malloch D 1970 New concepts in the Microascaceae illustrated by two new species. Mycologia. 62:727–740. doi:10.2307/3757662

[CIT0101] Matsumoto M, Asaoka T, Nagaoka K, Yokoi K, Nakajima T 1986 Novel antibiotic SS19508D and preparation thereof JPS 6143182 (A). Tokyo: S.S. Pharmaceutical Co. Ltd.

[CIT0102] Miller KI, Qing C, Sze DMY, Neilan BA, Cho WCS 2012 Investigation of the biosynthetic potential of endophytes in traditional Chinese anticancer herbs. PloS ONE. 7:e35953. doi:10.1371/journal.pone.0035953 22629306 PMC3358349

[CIT0103] Miller KI, Qing C, Sze DMY, Roufogalis BD, Neilan BA 2012 Culturable endophytes of medicinal plants and the genetic basis for their bioactivity. Microb Ecol. 64:431–449. doi:10.1007/S00248-012-0044-8 22430508

[CIT0104] Miller MA, Pfeiffer W, Schwartz T 2010 Creating the CIPRES Science Gateway for inference of large phylogenetic trees. Paper presented at: Proceedings of the Gateway Computing Environments Workshop (GCE); 1114; New Orleans, LA.

[CIT0105] Mishra A, Gond SK, Kumar A, Sharma VK, Verma SK, Kharwar RN, Sieber TN 2012 Season and tissue type affect fungal endophyte communities of the Indian medicinal plant *Tinospora cordifolia* more strongly than geographic location. Microb Ecol. 64:388–398. doi:10.1007/s00248-012-0029-7 22430503

[CIT0106] Morishima C, Shuhart MC, Wang CC, Paschal DM, Apodaca MC, Liu YZ, Sloan DD, Graf TN, Oberlies NH, Lee DYW, et al. 2010 Silymarin inhibits in vitro T-cell proliferation and cytokine production in hepatitis C virus infection. Gastroenterology. 138:671–681. doi:10.1053/J.Gastro.2009.09.021 19782083 PMC2819600

[CIT0107] Mouafo Talontsi F, Kongue Tatong MD, Dittrich B, Douanla-Meli C, Laatsch H 2013 Structures and absolute configuration of three α-pyrones from an endophytic fungus *Aspergillus niger*. Tetrahedron. 69:7147–7151. doi:10.1016/j.tet.2013.05.098

[CIT0108] Naik SB, Shashikala J, Krishnamurthy YL 2008 Diversity of fungal endophytes in shrubby medicinal plants of Malnad region, Western Ghats, Southern India. Fungal Ecol. 1:89–93. doi:10.1016/j.funeco.2008.05.001

[CIT0109] Namikoshi M, Kobayashi H, Yoshimoto T, Meguro S, Akano K 2000 Isolation and characterization of bioactive metabolites from marine-derived filamentous fungi collected from tropical and sub-tropical coral reefs. Chem Pharm Bull. 48:1452–1457. doi:10.1248/cpb.48.1452 11045449

[CIT0110] Napolitano JG, Lankin DC, Graf TN, Friesen JB, Chen S-N, McAlpine JB, Oberlies NH, Pauli GF 2013 HiFSA fingerprinting applied to isomers with near-identical NMR spectra: the Silybin/Isosilybin case. J Org Chem. 78:2827–2839. doi:10.1021/Jo302720h 23461697 PMC3640553

[CIT0111] Nilsson RH, Kristiansson E, Ryberg M, Hallenberg N, Larsson KH 2008 Intraspecific ITS variability in the kingdom fungi as expressed in the international sequence databases and its implications for molecular species identification. Evol Bioinform. 4:193–201. 10.4137/ebo.s653 PMC2614188 19204817

[CIT0112] Nilsson RH, Ryberg M, Kristiansson E, Abarenkov K, Larsson K-H, Koljalg U, Fairhead C 2006 Taxonomic reliability of DNA sequences in public sequence databases: a fungal perspective. PLoS ONE. 1:e59. doi:10.1371/journal.pone.0000059 17183689 PMC1762357

[CIT0113] Nilsson RH, Tedersoo L, Abarenkov K, Ryberg M, Kristiansson E, Hartmann M, Schoch CL, Nylander JAA, Bergsten J, Porter TM, et al. 2012 Five simple guidelines for establishing basic authenticity and reliability of newly generated fungal ITS sequences. Mycokeys. 4:37–63. doi:10.3897/mycokeys.4.3606

[CIT0114] Nishida H, Tomoda H, Cao J, Okuda S, Omura S 1991 Purpactins, new inhibitors of acyl-CoA: cholesterol acyltransferase produced by *Penicillium purpurogenum*. II. Structure elucidation of purpactins A, B and C. J Antibiot. 44:144–151. doi:10.7164/antibiotics.44.144 2010354

[CIT0115] Oh H, Kwon TO, Gloer JB, Marvanová L, Shearer CA 1999 Tenellic acids A–D: new bioactive diphenyl ether derivatives from the aquatic fungus *Dendrospora tenella*. J Nat Prod. 62:580–583. doi:10.1021/np980496m 10217713

[CIT0116] Okuno T, Oikawa S, Goto T, Sawai K, Shirahama H, Matsumoto T 1986 Structures and phytotoxicity of metabolites from *Valsa ceratosperma*. Agric Biol Chem. 50:997–1001. doi:10.1271/bbb1961.50.997

[CIT0117] Pažoutová S, Follert S, Bitzer J, Keck M, Surup F, Šrůtka P, Holuša J, Stadler M 2013 A new endophytic insect-associated *Daldinia* species, recognised from a comparison of secondary metabolite profiles and molecular phylogeny. Fung Divers. 60:107–123. doi:10.1007/s13225-013-0238-5

[CIT0118] Petrini O 1991 Fungal endophytes in tree leaves In: Andrews JH, Hirano SS, editors. Microbial ecology of leaves. doi:10.1007/978-1-4612-3168-4_9

[CIT0119] Polyak SJ, Morishima C, Lohmann V, Pal S, Lee DYW, Liu YZ, Graf TN, Oberlies NH 2010 Identification of hepatoprotective flavonolignans from silymarin. Proc Natl Acad Sci USA. 107:5995–5999. doi:10.1073/Pnas.0914009107 20231449 PMC2851903

[CIT0120] Polyak SJ, Oberlies NH, Pécheur EI, Dahari H, Ferenci P, Pawlotsky JM 2013 Silymarin for HCV infection. Antivir Ther. 18:141–147. doi:10.3851/IMP2402 23011959 PMC4076489

[CIT0121] Promputtha I, Miller AN 2010 Three new species of *Acanthostigma* (Tubeufiaceae, Dothideomycetes) from Great Smoky Mountains National Park. Mycologia. 102:574–587. doi:10.3852/09-051 20524590

[CIT0122] Puri SC, Verma V, Amna T, Qazi GN, Spiteller M 2005 An endophytic fungus from *Nothapodytes foetida* that produces camptothecin. J Nat Prod. 68:1717–1719. doi:10.1021/np0502802 16378360

[CIT0123] Qin J-C, Zhang Y-M, Gao J-M, Bai M-S, Yang S-X, Laatsch H, Zhang A-L 2009 Bioactive metabolites produced by *Chaetomium globosum*, an endophytic fungus isolated from *Ginkgo biloba*. Bioorg Med Chem Lett. 19:1572–1574. doi:10.1016/j.bmcl.2009.02.025 19246197

[CIT0124] Rehner SA, Samuels GJ 1995 Molecular systematics of the Hypocreales: a teleomorph gene phylogeny and the status of their anamorphs. Can J Bot. 73:S816–S823. doi:10.1139/b95-327

[CIT0125] Rodriguez RJ, White JF Jr, Arnold AE, Redman RS 2009 Fungal endophytes: diversity and functional roles: Tansley review. New Phytol. 182:314–330. doi:10.1111/j.1469-8137.2009.02773.x 19236579

[CIT0126] Romero A, Carrión G, Rico-Gray V 2001 Fungal latent pathogens and endophytes from leaves of *Parthenium hysterophorus* (Asteraceae). Fung Divers. 7:81–87.

[CIT0127] Saikkonen K 2007 Forest structure and fungal endophytes. Fungal Biol Rev. 21:67–74. doi:10.1016/j.fbr.2007.05.001

[CIT0128] Saikkonen K, Faeth SH, Helander M, Sullivan TJ 1998 Fungal endophytes: a continuum of interactions with host plants. Annu Rev Ecol Syst. 29:319–343. doi:10.1146/annurev.ecolsys.29.1.319

[CIT0129] Sánchez-Ballesteros J, González V, Salazar O, Acero J, Portal MA, Julián M, Rubio V, Bills GF, Polishook JD, Platas G, et al. 2000 Phylogenetic study of *Hypoxylon* and related genera based on ribosomal ITS sequences. Mycologia. 92:964–977. doi:10.2307/3761591

[CIT0130] Sassa T, Takemura T, Ikeda M, Miura Y 1973 Structure of radiclonic acid, a new plant growth-regulator produced by a fungus. Tetrahedron Lett. 14:2333–2334. doi:10.1016/S0040-4039(01)96211-2

[CIT0131] Sato S, Okusa N, Ogawa A, Ikenoue T, Seki T, Tsuji T 2005 Identification and preliminary SAR studies of (+)-geodin as a glucose uptake stimulator for rat adipocytes. J Antibiot. 58:583–589. doi:10.1038/ja.2005.79 16320762

[CIT0132] Schmeda-Hirschmann G, Hormazabal E, Rodriguez JA, Theoduloz C 2008 Cycloaspeptide A and pseurotin A from the endophytic fungus *Penicillium janczewskii*. Z Naturforsch C. 63:383–388. 18669024 10.1515/znc-2008-5-612

[CIT0133] Schoch CL, Seifert KA, Huhndorf S, Robert V, Spouge JL, Levesque CA, Chen W, Fungal Barcoding C 2012 Nuclear ribosomal internal transcribed spacer (ITS) region as a universal DNA barcode marker for fungi. Proc Natl Acad Sci USA. 109:6241–6246. doi:10.1073/pnas.1117018109 22454494 PMC3341068

[CIT0134] Schoch CL, Sung G-H, Lopez-Giraldez F, Townsend JP, Miadlikowska J, Hofstetter V, Robbertse B, Matheny PB, Kauff F, Wang Z, et al. 2009 The Ascomycota tree of life: a phylum-wide phylogeny clarifies the origin and evolution of fundamental reproductive and ecological traits. Syst Biol. 58:224–239. doi:10.1093/Sysbio/Syp020 20525580

[CIT0135] Schulz B, Boyle C, Draeger S, Römmert A-K, Krohn K 2002 Endophytic fungi: a source of novel biologically active secondary metabolites. Mycol Res. 106:996–1004. doi:10.1017/S0953756202006342

[CIT0136] Seto H, Sasaki T, Yonehara H 1977 Studies on the biosynthesis of radiclonic acid. Tetrahedron Lett. 18:4083–4084. doi:10.1016/S0040-4039(01)83432-8

[CIT0137] Shaaban M, Shaaban KA, Abdel-Aziz MS 2012 Seven naphtho-γ-pyrones from the marine-derived fungus *Alternaria alternata*: structure elucidation and biological properties. Org Med Chem Lett. 2:1–8. doi:10.1186/2191-2858-2-6 22377027 PMC3350997

[CIT0138] Shipunov A, Newcombe G, Raghavendra AKH, Anderson CL 2008 Hidden diversity of endophytic fungi in an invasive plant. Am J Bot. 95:1096–1108. doi:10.3732/ajb.0800024 21632429

[CIT0139] Shultz B, Guske S, Dammann U, Boyle C 1998 Endophytes–host interactions II. Defining symbiosis of endophytes–host interaction. Symbiosis. 25:213–227.

[CIT0140] Smith SA, Tank DC, Boulanger L-A, Bascom-Slack CA, Eisenman K, Kingery D, Babbs B, Fenn K, Greene JS, Hann BD, et al. 2008 Bioactive endophytes warrant intensified exploration and conservation. PLoS ONE. 3:e3052. doi:10.1371/journal.pone.0003052 18725962 PMC2518837

[CIT0141] Solfrizzo M, Visconti A, Savard M, Blackwell B, Nelson P 1994 Isolation and characterization of new chlamydosporol related metabolites of *Fusarium chlamydosporum* and *Fusarium tricinctum*. Mycopathologia. 127:95–101. doi:10.1007/BF01103065 7984219

[CIT0142] Soytong K, Kanokmedhakul S, Kukongviriyapan V, Isobe M 2001 Application of *Chaetomium* species (Ketomium®) as a new broad spectrum biological fungicide for plant disease con trol: a review article. Fung Divers. 7:1–15.

[CIT0143] Stamatakis A, Hoover P, Rougemont J 2008 A rapid bootstrap algorithm for the RAxML web servers. Syst Biol. 57:758–771. doi:10.1080/10635150802429642 18853362

[CIT0144] Steyn P 1973 The structures of five diketopiperazines from *Aspergillus ustus*. Tetrahedron. 29:107–120. doi:10.1016/S0040-4020(01)99384-6

[CIT0145] Stone JK, Bacon CW, White JF Jr 2000 An overview of endophytics microbes: endophytism defined In: Bacon CW, White JF Jr, editors. Microbial endophytes. New York (NY): Marcel Dekker; p. 3–30.

[CIT0146] Strobel G, Daisy B 2003 Bioprospecting for microbial endophytes and their natural products. Microbiol Mol Biol Rev. 67:491–502. doi:10.1128/mmbr.67.4.491-502.2003 14665674 PMC309047

[CIT0147] Strobel G, Daisy B, Castillo U, Harper J 2004 Natural products from endophytic microorganisms. J Nat Prod. 67:257–268. doi:10.1021/np030397v 14987067

[CIT0148] Strobel SA, Strobel GA 2007 Plant endophytes as a platform for discovery-based undergraduate science education. Nat Chem Biol. 3:356–359. doi:10.1038/Nchembio0707-356 17576416

[CIT0149] Sumarah MW, Puniani E, Blackwell BA, Miller JD 2008 Characterization of polyketide metabolites from foliar endophytes of *Picea glauca*. J Nat Prod. 71:1393–1398. doi:10.1021/np800192f 18636777

[CIT0150] Suryanarayanan TS, Thirunavukkarasu N, Govindarajulu MB, Sasse F, Jansen R, Murali TS 2009 Fungal endophytes and bioprospecting. Fungal Biol Rev. 23:9–19. doi:10.1016/j.fbr.2009.07.001

[CIT0151] Suryanarayanan TS, Wittlinger SK, Faeth SH 2005 Endophytic fungi associated with cacti in Arizona. Mycol Res. 109:635–639. doi:10.1017/S0953756205002753 16018319

[CIT0152] Sy-Cordero AA, Day CS, Oberlies NH 2012 Absolute configuration of isosilybin A by X-ray crystallography of the heavy atom analogue 7-(4-bromobenzoyl)isosilybin A. J Nat Prod. 75:1879–1881. doi:10.1021/np3005369 23116206 PMC3620721

[CIT0153] Syed NA, Midgley DJ, Ly PKC, Saleeba JA, McGee PA 2009 Do plant endophytic and free–living *Chaetomium* species differ? Aust Mycol. 28:51–55.

[CIT0154] Tae HS, Hines J, Schneekloth AR, Crews CM 2010 Total synthesis and biological evaluation of tyroscherin. Org Lett. 12:4308–4311. doi:10.1021/ol101801u 20831175 PMC3175621

[CIT0155] Talavera G, Castresana J 2007 Improvement of phylogenies after removing divergent and ambiguously aligned blocks from protein sequence alignments. Syst Biol. 56:564–577. doi:10.1080/10635150701472164 17654362

[CIT0156] Tamm C, Böhner B, Zürcher W 1972 Myrochromanol und myrochromanon, zwei weitere metaboliten von *Myrothecium roridum* Tode ex Fr. Verrucarine und Roridine, 25. Mitt Helv Chim Acta. 55:510–518. doi:10.1002/hlca.19720550222

[CIT0157] Tan RX, Zou WX 2001 Endophytes: a rich source of functional metabolites (1987 to 2000). Nat Prod Rep. 18:448–459. doi:10.1039/B100918o 11548053

[CIT0158] Tanaka Y, Matsuzaki K, Zhong C-L, Yoshida H, Kawakubo T, Masuma R, Tanaka H, Omura S 1996 Dechlorogeodin and its new dihydro derivatives, fungal metabolites with herbicidal activity. J Antibiot. 49:1056–1059. doi:10.7164/antibiotics.49.1056 8968402

[CIT0159] Taylor JE, Hyde KD, Jones EBG 1999 Endophytic fungi associated with the temperate palm, *Trachycarpus fortunei*, within and outside its natural geographic range. New Phytol. 142:335–346. doi:10.1046/j.1469-8137.1999.00391.x

[CIT0160] Tayone WC, Honma M, Kanamaru S, Noguchi S, Tanaka K, Nehira T, Hashimoto M 2011 Stereochemical investigations of isochromenones and isobenzofuranones isolated from *Leptosphaeria* sp. KTC 727. J Nat Prod. 74:425–429. doi:10.1021/np100838j 21265556

[CIT0161] Tejesvi MV, Kajula M, Mattila S, Pirttila AM 2011 Bioactivity and genetic diversity of endophytic fungi in *Rhododendron tomentosum* Harmaja. Fung Divers. 47:97–107. doi:10.1007/S13225-010-0087-4.

[CIT0162] Tejesvi MV, Kini KR, Prakash HS, Subbiah V, Shetty HS 2007 Genetic diversity and antifungal activity of species of *Pestalotiopsis* isolated as endophytes from medicinal plants. Fung Divers. 24:37–54.

[CIT0163] Tejesvi MV, Mahesh B, Nalini MS, Prakash HS, Kini KR, Subbiah V, Shetty HS 2006 Fungal endophyte assemblages from ethnopharmaceutically important medicinal trees. Can J Microbiol. 52:427–435. doi:10.1139/w05-143 16699567

[CIT0164] Tomoda H, Huang XH, Cao J, Nishida H, Nagao R, Okuda S, Tanaka H, Omura S, Arai H, Inoue K 1992 Inhibition of acyl-CoA: cholesterol acyltransferase activity by cyclodepsiptide antibiotics. J Antibiot. 45:1626–1632. doi:10.7164/antibiotics.45.1626 1473990

[CIT0165] Trifonov L, Hilpert H, Floersheim P, Dreiding A, Rast D, Skrivanova R, Hoesch L 1986 Bisvertinols: a new group of dimeric vertinoids from *Verticillium intertextum*. Tetrahedron. 42:3157–3179. doi:10.1016/S0040-4020(01)87382-8

[CIT0166] VanderMolen KM, Raja HA, El-Elimat T, Oberlies NH 2013 Evaluation of culture media for the production of secondary metabolites in a natural products screening program. AMB Express. 3:71. doi:10.1186/2191-0855-3-71 24342059 PMC3917616

[CIT0167] Varoglu M, Corbett TH, Valeriote FA, Crews P 1997 Asperazine, a selective cytotoxic alkaloid from a sponge-derived culture of *Aspergillus niger*. J Org Chem. 62:7078–7079. doi:10.1021/jo970568z 11671801

[CIT0168] Verma VC, Lobkovsky E, Gange AC, Singh SK, Prakash S 2011 Piperine production by endophytic fungus *Periconia* sp. isolated from *Piper longum* L. J Antibiot. 64:427–431. doi:10.1038/ja.2011.27. 21505472

[CIT0169] Vieira MLA, Hughes AFS, Gil VB, Vaz ABM, Alves TMA, Zani CL, Rosa CA, Rosa LH 2012 Diversity and antimicrobial activities of the fungal endophyte community associated with the traditional Brazilian medicinal plant *Solanum cernuum* vell. (Solanaceae). Can J Microbiol. 58:54–66. doi:10.1139/w11-105 22182199

[CIT0170] Vilgalys R, Hester M 1990 Rapid genetic identification and mapping of enzymatically amplified ribosomal DNA from several *Cryptococcus* species. J Bacteriol. 172:4238–4246. 2376561 10.1128/jb.172.8.4238-4246.1990 PMC213247

[CIT0171] Visconti A, Solfrizzo M, Fruchier A, ApSimon JW 1994 Acuminatopyrone: revised structure and production by *Fusarium chlamydosporum* and *Fusarium tricinctum*. J Nat Prod. 57:695–699. doi:10.1021/np50108a002

[CIT0172] Wagoner J, Morishima C, Graf TN, Oberlies NH, Teissier E, Pécheur E-I, Tavis JE, Polyak SJ, Mossman K 2011 Differential in vitro effects of intravenous versus oral formulations of Silibinin on the HCV life cycle and inflammation. PloS ONE. 6:e16464. doi:10.1371/journal.pone.0016464 21297992 PMC3030583

[CIT0173] Wagoner J, Negash A, Kane OJ, Martinez LE, Nahmias Y, Bourne N, Owen DM, Grove J, Brimacombe C, McKeating JA, et al. 2010 Multiple effects of Silymarin on the Hepatitis C virus lifecycle. Hepatology. 51:1912–1921. doi:10.1002/Hep.23587 20512985 PMC2909978

[CIT0174] Wang J, Huang Y, Fang M, Zhang Y, Zheng Z, Zhao Y, Su W 2002 Brefeldin A, a cytotoxin produced by *Paecilomyces* sp. and *Aspergillus clavatus* isolated from *Taxus mairei* and *Torreya grandis*. FEMS Immunol Med Microbiol. 34:51–57. doi:10.1111/j.1574-695X.2002.tb00602.x 12208606

[CIT0175] Wang Q, Xu L 2012 Beauvericin, a bioactive compound produced by fungi: a short review. Molecules. 17:2367–2377. doi:10.3390/molecules17032367 22367030 PMC6269041

[CIT0176] Wen L, Chen G, She Z, Yan C, Cai J, Mu L 2010 Two new paeciloxocins from a mangrove endophytic fungus *Paecilomyces* sp. Russ Chem Bull. 59:1656–1659. doi:10.1007/s11172-010-0290-1

[CIT0177] White TJ, Bruns T, Lee SB, Taylor JW 1990 Amplification and direct sequencing of fungal ribosomal RNA genes for phylogenetics In: Innis MA, Gefland DH, Sninsky JJ, White TJ, editors. PCR protocols: a guide to methods and application. San Diego (CA): Academic Press; p. 315–322.

[CIT0178] Wilson D 1995 Endophyte: the evolution of a term, and clarification of its use and definition. Oikos. 73:274–276. doi:10.2307/3545919

[CIT0179] Xu L, Wang J, Zhao J, Li P, Shan T, Li X, Zhou L 2010 Beauvericin from the endophytic fungus, *Fusarium redolens*, isolated from *Dioscorea zingiberensis* and its antibacterial activity. Nat Prod Commun. 5:811–814. doi:10.1016/j.jbiotec.2008.07.304. 20521553

[CIT0180] Yang Y, Lowry M, Xu X, Escobedo JO, Sibrian-Vazquez M, Wong L, Schowalter CM, Jensen TJ, Fronczek FR, Warner IM 2008 Seminaphthofluorones are a family of water-soluble, low molecular weight, NIR-emitting fluorophores. Proc Natl Acad Sci USA. 105:8829–8834. doi:10.1073/pnas.0710341105 18579790 PMC2440362

[CIT0181] Zhan J, Burns AM, Liu MX, Faeth SH, Gunatilaka AAL 2007 Search for cell motility and angiogenesis inhibitors with potential anticancer activity: beauvericin and other constituents of two endophytic strains of *Fusarium oxysporum*. J Nat Prod. 70:227–232. doi:10.1021/np060394t 17286429 PMC3361905

[CIT0182] Zhang Y, Zhu T, Fang Y, Liu H, Gu Q, Zhu W 2007 Carbonarones A and B, new bioactive γ-pyrone and α-pyridone derivatives from the marine-derived fungus *Aspergillus carbonarius*. J Antibiot. 60:153–157. doi:10.1038/ja.2007.15 17420566

[CIT0183] Zhao K, Penttinen P, Guan TW, Xiao J, Chen QA, Xu J, Lindstrom K, Zhang LL, Zhang XP, Strobel GA 2011 The diversity and anti-microbial activity of endophytic Actinomycetes isolated from medicinal plants in Panxi plateau, China. Curr Microbiol. 62:182–190. doi:10.1007/S00284-010-9685-3 20567975

[CIT0184] Zubek S, Błaszkowski J, Buchwald W 2012 Fungal root endophyte associations of medicinal plants. Nova Hedwig. 94:525–540. doi:10.1127/0029-5035/2012/0024

