# Supplementary material for: Phylogenetic and chemical diversity of fungal endophytes isolated from Silybum marianum (L) Gaertn. (milk thistle)
Source: Mycology. 2015 Feb 23;6(1):8–27. doi: 10.1080/21501203.2015.1009186 (PMC4409047; doi:10.1080/21501203.2015.1009186)
Supplement: Supplementary_material.docx [file TMYC_A_1009186_SM7119.docx]

Phylogenetic and Chemical Diversity of Fungal Endophytes Isolated from *Silybum marianum* (L) Gaertn. (Milk Thistle)

Huzefa A. Raja^a,†^, Amninder Kaur^a,†^, Tamam El-Elimat^a^, Mario Figueroa^b^, Rahul Kumar^c^, Gagan Deep^c^, Rajesh Agarwal^c^, Stanley H. Faeth^d^, Nadja B. Cech^a^, and Nicholas H. Oberlies^a,*^

^a^Department of Chemistry and Biochemistry, University of North Carolina at Greensboro, Greensboro, NC 27402, United States

^b^Facultad de Química, Universidad Nacional Autónoma de México, Mexico DF 04510, Mexico

^c^Department of Pharmaceutical Sciences, Skaggs School of Pharmacy and Pharmaceutical Sciences, University of Colorado Denver, Aurora, CO 80045, United States

^d^Department of Biology, University of North Carolina at Greensboro, NC 27402, United States

^*^Corresponding author. Tel.: +1 3363345474.

E-mail address: nicholas_oberlies@uncg.edu (N.H. Oberlies).

^†^These authors contributed equally to this work.

**List of Supplementary Information Figures**:

S1. Chemical structures, high-resolution MS, and selected NMR data for the secondary metabolites isolated and/or identified from the endophytic fungal extracts of milk thistle

S2. ^1^H NMR (400 MHz; top) and ^13^C NMR (100 MHz; bottom) spectra of biscognin A (**1**) in CDCl_3_

S3. ^1^H NMR (400 MHz; top) and ^13^C NMR (100 MHz; bottom) spectra of biscognin B (**2**) in CDCl_3_

S4. ^1^H NMR (400 MHz; top) and ^13^C NMR (175 MHz; bottom) spectra of monascuskaoliaone B (**4**) in CDCl_3_

S5. Observed chemical shift differences (Δ*δ* = *δ*_S_ − *δ*_R_, ppm; 500 MHz) for the *R*- and *S*-MTPA esters of (a) biscognin A (**1**) and (b) monascuskaoliaone B (**4**)

S6. (**a**) Conformational analysis, (**b**) ECD spectra (experimental and calculated), and (**c**) specific rotation values (experimental and calculated) of monascuskaoliaone B (**4**), monascuskaoliaone (**5**), and their corresponding C-2 epimer and enantiomer, respectively, in MeOH using DFT B3LYP/DGDZVP

S7. Phylogram of the most likely tree (-lnL = 5345.50) from a RAxML analysis of 41 isolates based on complete ITS rDNA (436 bp). Numbers refer to RAxML bootstrap support values ≥ 70% based on 1000 replicates. Bar indicates nucleotide substitution per site. For each OTU, the plant tissue type is indicated on the right.

S8. Phylogram of the most likely tree (-lnL = 27826.94) from a RAxML analysis of 226 isolates based on partial LSU rDNA (1264 bp). Numbers refer to RAxML bootstrap support values ≥ 70% based on 1000 replicates. Fungal endophytes from milk thistle are highlighted in bold. Classification following (Lumbsch and Huhndorf 2010) is shown on the right. Bar indicates nucleotide substitution per site.

S9. Effect of selected fungal metabolites on the viability of human prostate carcinoma (PC-3) cells

- Preparation of Mosher’s esters of biscognin A (**1**) and monascuskaoliaone B (**4**)
- Computational methods

S1: Chemical structures, high-resolution MS, and selected NMR data for the secondary metabolites isolated and/or identified from the endophytic fungal extracts of milk thistle.

*In a few cases where complete NMR data for the compounds encountered in this study were not reported in literature, these data have been presented below. In the process of structure elucidation, if the NMR data for selected compounds were recorded in additional deuterated solvents (other than those reported in literature), we also updated these additional NMR data.*

| **#** | **Compounds** | **Structures** | **HRMS Data [Calculated]** | **NMR** |
| --- | --- | --- | --- | --- |
| 1 | Alternariol |  | 259.0589 (M+H)^+^  [259.0601; calculated for C_14_H_11_O_5_;  Δ= ‒4.6 ppm] | NMR data were fully consistent with those reported in literature  (Koch et al. 2005) |
| 2 | 9-*o*-methylalternariol |  | 273.0744 (M+H)^+^  [273.0758; calculated for C_15_H_13_O_5_;  Δ= ‒4.9 ppm] | NMR data were fully consistent with those reported in literature  (de Souza et al. 2013) |
| 3 | Destruxin B |  | 594.3833 (M+H)^+^  [594.3861; calculated for C_30_H_52_N_5_O_7_;  Δ= ‒4.8 ppm] | NMR data were fully consistent with those reported in literature  (Buchwaldt and Jensen 1991) |
| 4 | Homodestruxin B |  | 608.3992 (M+H)^+^  [608.4018; calculated for C_31_H_54_N_5_O_7_;  Δ= ‒4.2 ppm] | NMR data were fully consistent with those reported in literature  (Buchwaldt and Jensen 1991) |
| 5 | Antibiotic PF 1052 |  | 430.2942 (M+H)^+^  [430.2952; calculated for C_26_H_40_NO_4_;  Δ= ‒2.3 ppm] | NMR data were fully consistent with those reported in literature  (Koyama et al. 2005) |
| 6 | Euplectin |  | 295.0595 (M+H)^+^  [295.0601; calculated for C_17_H_11_O_5_;  Δ= ‒2.0 ppm] | NMR data were fully consistent with those reported in literature  (Ernst-Russell et al. 1999) |
| 7 | Coneuplectin |  | 297.0749 (M+H)^+^  [297.0758; calculated for C_17_H_13_O_5_;  Δ= ‒2.8 ppm] | NMR data were fully consistent with those reported in literature  (Ernst-Russell et al. 1999) |
| 8 | Pyrenocine A |  | 209.0803 (M+H)^+^  [209.0808; calculated for C_11_H_13_O_4_;  Δ= ‒2.6 ppm] | NMR data were fully consistent with those reported in literature  (Hashida et al. 2010) |
| 9 | Pyrenocine B |  | 227.0908 (M+H)^+^  [227.0914; calculated for C_11_H_15_O_5_;  Δ= ‒2.6 ppm] | NMR data were fully consistent with those reported in literature  (Hashida et al. 2010) |
| 10 | *R*-7-hydroxy-3-(*S*-1-hydroxyethyl)-5-methoxy-3,4-dimethylisobenzofuran-1(3H)-one |  | 253.1065 (M+H)^+^  [253.1071; calculated for C_13_H_17_O_5_;  Δ= ‒2.2 ppm] | NMR data were fully consistent with those reported in literature  (Tayone et al. 2011) |
| 11 | *R*-4,8-dihydroxy-6-methoxy-4,5-dimethyl-3-methyleneisochroman-1-one |  | 251.0908 (M+H)^+^  [251.0914; calculated for C_13_H_15_O_5_;  Δ= ‒2.4 ppm] | NMR data were fully consistent with those reported in literature  (Tayone et al. 2011) |
| 12 | Methyl asterrate/ Trimethylosoic acid |  | 363.1069 (M+H)^+^  [363.1074; calculated for C_18_H_19_O_8_;  Δ= ‒1.5 ppm] | NMR data were fully consistent with those reported in literature  (Hargreaves et al. 2002b) |
| 13 | Methyl 2,4-dichloroasterrate |  | 431.0281 (M+H)^+^  [431.0295; calculated for C_18_H_17_O_8_Cl_2_;  Δ= ‒3.2 ppm] | NMR data were fully consistent with those reported in literature  (Lin et al. 2009) |
| 14 | Dihydrogeodin |  | 401.0184 (M+H)^+^  [401.0189; calculated for C_17_H_15_O_7_Cl_2_;  Δ= ‒1.3 ppm] | NMR data were fully consistent with those reported in literature  (Sato et al. 2005) |
| 15 | Bisdechlorogeodin |  | 331.0806 (M+H)^+^  [331.0812; calculated for C_17_H_15_O_7_;  Δ= ‒1.8 ppm] | NMR data were fully consistent with those reported in literature  (Tanaka et al. 1996) |
| 16 | Antibiotic SS 19508D |  | 365.0416 (M+H)^+^  [365.0404; calculated for C_17_H_14_O_7_Cl;  Δ= ‒1.9 ppm] | NMR data were fully consistent with those reported in literature  (Matsumoto et al. 1986) |
| 17 | Geodin |  | 399.0024 (M+H)^+^  [399.0033; calculated for C_17_H_13_O_7_Cl_2_;  Δ= ‒2.2 ppm] | NMR data were fully consistent with those reported in literature  (Hargreaves et al. 2002b) |
| 18 | (-)-α-tetrahydro-bisabolen-2,5,6-triol |  | 257.2118 (M+H)^+^  [257.2111; calculated for C_15_H_29_O_3_;  Δ= +2.7 ppm] | Identified by UPLC-PDA-HRMS-MS/MS dereplication protocol  (El-Elimat et al. 2013) |
| 19 | Thielavin B |  | 567.2224 (M+H)^+^  [567.2230; calculated for C_31_H_35_O_10_;  Δ= ‒1.1 ppm] | NMR data were fully consistent with those reported in literature  (Jang et al. 2014)  The structure was also confirmed by MSMS and 2D NMR data. |
| 20 | Thielavin C |  | 579.2218 (M‒H)^‒^  [579.2230; calculated for C_32_H_35_O_10_;  Δ= ‒2.1 ppm] | White powder; ^1^H NMR data (CD_3_OD; 400 MHz) *δ* 3.83 (s,2-OMe), 3.82 (s, 2’-OMe), 2.65 (s, 6”- Me), 2.41 (s, 6-Me), 2.29 (s, 6’-Me), 2.26 (s, 3’-Me), 2.25 (s, 5’-Me), 2.21 (s, 5”- Me), 2.18 (s, 3-Me), 2.14 (s, 5-Me), 2.12 (s, 3”- Me),  The structure was also confirmed by MSMS and 2D NMR data. |
| 21 | Bisorbicillinolide |  | 495.2038 (M‒H)^‒^  [495.2024; calculated for C_28_H_31_O_8_;  Δ= +2.7 ppm] | NMR data were fully consistent with those reported in literature  (Abe et al. 1998) |
| 22 | Bisvertinolone |  | 513.2109 (M+H)^+^  [513.2119; calculated for C_28_H_33_O_9_;  Δ= ‒2.0 ppm] | NMR data were fully consistent with those reported in literature  (Trifonov et al. 1986) |
| 23 | Trichodimerol |  | 495.2037 (M‒H)^‒^  [495.2024; calculated for C_28_H_31_O_8_;  Δ= +2.5 ppm] | NMR data were fully consistent with those reported in literature  (Andrade et al. 1992) |
| 24 | Verrucarin A |  | 503.2244 (M+H)^+^  [503.2276; calculated for C_27_H_35_O_9_;  Δ= ‒6.3 ppm] | NMR data were fully consistent with those reported in literature  (Liu et al. 2006) |
| 25 | Verrucarin J |  | 485.2140 (M+H)^+^  [485.2170; calculated for C_27_H_33_O_8_;  Δ= ‒6.2 ppm] | NMR data were fully consistent with those reported in literature  (Namikoshi et al. 2000) |
| 26 | Verrucarin L acetate |  | 543.2225 (M+H)^+^  [543.2225; calculated for C_29_H_35_O_10_;  Δ= 0.0 ppm] | NMR data were fully consistent with those reported in literature  (Namikoshi et al. 2000) |
| 27 | Myrochromanol |  | 187.1117 (M+H‒H_2_O)^+^  [187.1117; calculated for C_13_H_15_O;  Δ= 0.0 ppm] | NMR data were fully consistent with those reported in literature  (Tamm et al. 1972) |
| 28 | Dihydro-5-(hydroxyphenylmethyl)-2(3H)-furanone |  | 193.0862 (M+H)^+^  [193.0859; calculated for C_11_H_13_O_3_;  Δ= +1.4 ppm] | NMR data were fully consistent with those reported in literature  (Hargreaves et al. 2002a) |
| 29 | Beauvericin |  | 784.4140 (M+H)^+^  [784.4168; calculated for C_45_H_58_N_3_O_9_;  Δ= ‒3.5 ppm] | NMR data were fully consistent with those reported in literature  (Xu et al. 2010) |
| 30 | Monascuskaoliaone |  | 283.2275 (M+H)^+^  [283.2268; calculated for C_17_H_31_O_3_;  Δ= +2.6 ppm] | Due to low sample amount, a credible optical rotation value could not be obtained. Instead ECD data were collected for this optically active sample and are discussed in Fig. S6 below.  NMR data were fully consistent with those reported in literature  (Cheng et al. 2010) |
| 31 | (3*R*)-5-Methylmellein |  | 193.0858 (M+H)^+^  [193.0859; calculated for C_11_H_13_O_3_;  Δ= ‒0.6 ppm] | NMR data and optical rotation values were consistent with those reported in literature (Okuno et al. 1986) |
| 32 | (3*R*)-5-Formylmellein |  | 207.0645 (M+H)^+^  [207.0652; calculated for C_11_H_11_O_4_;  Δ= ‒3.3 ppm] | NMR data were fully consistent with those reported in literature  (Sumarah et al. 2008) |
| 33 | (3*R*)-6-Methoxy-5-methylmellein |  | 223.0958 (M+H)^+^  [223.0965; calculated for C_12_H_15_O_4_;  Δ= ‒3.1 ppm] | Since the ^1^H NMR data did not completely match the data reported in literature (Anderson et al. 1983), a complete set of 2D NMR data were collected to validate the structure. The ^1^H and ^13^C NMR data are presented below.  White powder; [α]^25^_D_ ‒94  (*c* 0.45, CHCl_3_); UV/Vis (MeOH) λ_max_ (log *ε*) 233 (3.4), 271 (3.5), 313 (3.4) nm; ^1^H NMR data (CDCl_3_; 400 MHz) *δ* 11.35 (s, 8-OH), 6.35 (s, H-7), 4.59 (m, H-3), 3.83 (s, H_3_-11), 2.95 (dd, *J* = 16.5, 3.3, H-4_α_), 2.66 (dd, *J* = 16.5, 11.6, H-4_β_), 2.01 (s, H_3_-10), 1.51 (d, *J* = 6.4, H_3_-9); ^13^C NMR (CDCl_3_; 100 MHz) *δ* 170.7 (C-1), 164.3 (C-6), 163.1 (C-8), 138.0 (C-4a), 114.9 (C-5), 101.0 (C-8a), 97.6 (C-7), 75.1 (C-3), 55.9 (C-11), 32.3 (C-4), 21.1 (C-9), 10.7 (C-10). |
| 34 | Cyclo-[L-Phe-L-Leu-L-Leu-L-Leu-L-Ile] |  | 600.4102 (M+H)^+^  [600.4119; calculated for C_33_H_54_N_5_O_5_;  Δ= ‒2.9 ppm] | NMR data were fully consistent with those reported in literature  (Li et al. 2004) |
| 35 | (3*R*,4*R*)-*cis*-4-Hydroxy-5-methylmellein |  | 209.0803 (M+H)^+^  [209.0808; calculated for C_11_H_13_O_4_;  Δ= ‒2.6 ppm] | NMR data were fully consistent with those reported in literature  (Okuno et al. 1986) |
| 36 | Acuminstopyrone |  | 206.0817 (M+H)^+^  [206.0812; calculated for C_11_H_12_NO_3_;  Δ= +2.6 ppm] | NMR data were fully consistent with those reported in literature  (Visconti et al. 1994) |
| 37 | Chlamydospordiol |  | 229.1074 (M+H)^+^  [229.1071; calculated for C_11_H_17_O_5_;  Δ= +1.5 ppm] | NMR data were fully consistent with those reported in literature  (Solfrizzo et al. 1994) |
| 38 | Chlamydosporol (major:minor mixture) |  | 227.0914 (M+H)^+^  [229.0914; calculated for C_11_H_15_O_5_;  Δ= 0.0 ppm] | NMR data were fully consistent with those reported in literature  (Solfrizzo et al. 1994) |
| 39 | Tenellic acid C |  | 453.1507 (M+Na)^+^  [453.1520; calculated for C_23_H_26_O_8_Na;  Δ= ‒2.8 ppm] | NMR data were fully consistent with those reported in literature  (Oh et al. 1999) |
| 40 | Purpactin C |  | 435.1404 (M+Na)^+^  [453.1414; calculated for C_23_H_24_O_7_Na;  Δ= ‒2.4 ppm] | NMR data were fully consistent with those reported in literature  (Nishida et al. 1991) |
| 41 | Purpactin C’ |  | 435.1398 (M+Na)^+^  [435.1414; calculated for C_23_H_24_O_7_Na;  Δ= ‒3.7 ppm] | NMR data were fully consistent with those reported in literature  (Nishida et al. 1991) |
| 42 | Paeciloxocin A |  | 395.1457 (M+Na)^+^  [395.1465; calculated for C_21_H_24_O_6_Na;  Δ= ‒2.0 ppm] | NMR data were fully consistent with those reported in literature  (Wen et al. 2010) |
| 43 | Radiclonic acid |  | 397.2936 (M+H)^+^  [397.2949; calculated for C_23_H_41_O_5_;  Δ= ‒3.1 ppm] | NMR data for only the dimethyl ester derivative of radiclonic acid have been reported in literature (Sassa et al. 1973; Seto et al. 1977). Presented below are the ^1^H and ^13^C NMR data for the parent natural product.  ^1^H NMR data (CDCl_3_; 400 MHz) *δ* 6.11 (s, H-5), 5.20 (d, *J* = 9.6, H-7), 3.51 (dd, *J* = 10.2, 4.7, H_α_-21), 3.30 (dd, *J* = 10.2, 8.5, H_β_-21), 2.65 (m, H-2), 2.47 (m, H_2_-3), 2.47 (m, H-8), 1.73 (s, H_3_-19), 1.52 (m, H-12), 1.47 (m, H-10), 1.37 (m, H-14), 1.29 (m, H_α_-15), 1.18 (d, *J* = 6.3, H_3_-17), 1.17 (m, H_α_-13), 1.15 (m, H_α_-11), 1.08 (m, H_2_-9), 1.00 (m, H_β_-15), 0.97 (m, H_β_-11), 0.91 (d, *J* = 6.6, H_3_-20), 0.82 (overlapped, H_3_-22), 0.82 (m, H_β_-13), 0.81 (overlapped, H_3_-23), 0.81 (overlapped, H_3_-16); ^13^C NMR (CDCl_3_; 100 MHz) *δ* 181.5 (C-1), 174.1(C-18), 141.6 (C-5), 139.0 (C-7), 130.8 (C-6), 128.7 (C-4), 67.0 (C-21), 45.2 (C-13), 40.5 (C-11), 39.7 (C-2), 39.0 (C-3), 36.4 (C-10), 31.6 (C-14), 29.0 (C-15), 27.8 (C-12), 21.5 (C-20), 21.1 (C-22), 20.0 (C-23), 17.0 (C-17), 15.9 (C-19), 11.3 (C-16). |
| 44 | 10,20-Dehydro[12,13-dehydroprolyl-2-(1,1-dimethylallyl)tryptophyl]diketopiperazine] |  | 348.1691 (M+H)^+^  [348.1707; calculated for C_21_H_22_N_3_O_2_;  Δ= ‒4.5 ppm] | ^1^H NMR data were consistent with those reported in literature (Steyn 1973). Since ^13^C NMR data were not reported, these data as well as ^1^H NMR (400 MHz) data are presented below.  ^1^H NMR data (CDCl_3_; 400 MHz) *δ* 8.12 (br s, 1-NH), 7.42 (d, *J* = 7.7, H-4), 7.22 (d, *J* = 7.7, H-7), 7.05 (dt, *J* = 1.2, 7.7, H-6), 7.00 (dt, *J* = 1.2, 7.7, H-5), 5.78 (d, *J* = 8.7, H-19), 5.75 (d, *J* = 8.7, H-20), 5.44 (t, *J* = 3.0, H-13), 4.28 (d, *J* = 6.9, H-9), 3.60 (m, H_2_-15), 3.55 (m, H_α_-8), 3.38 (dd, *J* = 14.5, 6.9, H_β_-8), 2.21 (m, H_α_-14), 1.75 (m, H_β_-14), 1.62 (s, H_3_-21), 1.36 (s, H_3_-22); ^13^C NMR (CDCl_3_; 100 MHz) *δ* 162.6 (C-17), 156.5 (C-11), 142.2 (C-19), 141.2 (C-2), 134.8 (C-7a), 131.9 (C-12), 128.8 (C-3a), 121.6 (C-6), 121.1 (C-20), 119.8 (C-5), 119.0 (C-13), 117.8 (C-4), 110.7 (C-7), 104.1 (C-3), 60.8 (C-9), 45.7 (C-15), 37.9 (C-18), 32.4 (C-22), 27.5 (C-8), 27.4 (C-14), 25.4 (C-21). |
| 45 | 12,13-Dehydroprolyl-2-(1,1-dimethylallyltryptophyl)diketopiperazine |  | 350.1849 (M+H)^+^  [350.1863; calculated for C_21_H_24_N_3_O_2_;  Δ= ‒4.0 ppm] | ^1^H NMR data were consistent with those reported in literature  (Steyn 1973) |
| 46 | Deoxybrevianamide E |  | 352.2004 (M+H)^+^  [352.2020; calculated for C_21_H_26_N_3_O_2_;  Δ= ‒4.4 ppm] | NMR data were fully consistent with those reported in literature  (Steyn 1973) |
| 47 | Pseurotin A |  | 430.1497 (M–H)^–^  [430.1496; calculated for C_22_H_24_NO_8_;  Δ= +0.1 ppm] | NMR data were fully consistent with those reported in literature  (Schmeda-Hirschmann et al. 2008) |
| 48 | Tyroscherin |  | 334.2725 (M+H)^+^  [334.2740; calculated for C_21_H_36_NO_2_;  Δ= ‒4.7 ppm] | NMR data were fully consistent with those reported in literature  (Tae et al. 2010) |
| 49 | Asperazine |  | 665.2855 (M+H)^+^  [665.2871; calculated for C_40_H_37_N_6_O_4_;  Δ= ‒2.4 ppm] | NMR and ECD data were fully consistent with those reported in literature  (Varoglu et al. 1997) |
| 50 | Campyrone A |  | 254.1379 (M+H)^+^  [254.1386; calculated for C_13_H_20_NO_4_;  Δ= ‒3.1 ppm] | NMR data in CD_3_OD were fully consistent with those reported in literature (Mouafo Talontsi et al. 2013)  Colorless solid; ^1^H NMR data (CDCl_3_; 400 MHz) *δ* 6.00 (br d, *J* = 9.3, NH), 5.94 (d, *J* = 2.2, H-5), 5.41 (d, *J* = 2.2, H-3), 4.50 (t, *J* = 9.3, H-7), 3.78 (s, 4-OMe),1.99 (s, H_3_-2’), 1.85 (m, H-8), 1.55 (m, H-9_α_), 1.13 (m, H-9_β_), 0.88 (t, *J* = 7.4, H_3_-10), 0.83 (d, *J* = 6.9, H_3_-11); ^13^C NMR (CDCl_3_; 100 MHz) *δ* 171.2 (C-4), 169.9 (C-1’), 164.7 (C-2), 162.0 (C-6), 101.7 (C-5), 88.6 (C-3), 56.2 (4-OMe), 56.0 (C-7), 37.0 (C-8), 25.4 (C-9), 23.5 (C-2’), 15.9 (C-11), 11.2 (C-10). |
| 51 | Campyrone C |  | 240.1220 (M+H)^+^  [240.1230; calculated for C_12_H_18_NO_4_;  Δ= ‒4.3 ppm] | NMR data in CD_3_OD were fully consistent with those reported in literature (Mouafo Talontsi et al. 2013)  Colorless solid; ^1^H NMR data (CDCl_3_; 400 MHz) *δ* 6.10 (br d, *J* = 9.2, NH), 5.94 (d, *J* = 2.2, H-5), 5.41 (d, *J* = 2.2, H-3), 4.43 (t, *J* = 9.2, H-7), 3.78 (s, 4-OMe), 2.06 (m, H-8), 2.00 (s, H_3_-2’), 0.96 (d, *J* = 6.9, H_3_-9), 0.86 (d, *J* = 6.9, H_3_-10); ^13^C NMR (CDCl_3_; 100 MHz) *δ* 171.2 (C-4), 170.0 (C-1’), 164.6 (C-2), 162.0 (C-6), 101.6 (C-5), 88.6 (C-3), 57.3 (C-7), 56.2 (4-OMe), 30.9 (C-8), 23.4 (C-2’), 19.6 (C-10), 19.0 (C-9). |
| 52 | Fonsecin |  | 291.0854 (M+H)^+^  [291.0863; calculated for C_15_H_15_O_6_;  Δ= ‒3.1 ppm] | Yellow solid; ^1^H NMR data [(CD_3_)_2_CO; 400 MHz] *δ* 6.52 (br d, *J* = 1.9, H-9), 6.38 (br s, H-10), 6.36 (br d, *J* = 1.9, H-7), 3.88 (s, 6-OMe), 3.10 (d, *J* = 16.9, H-3_α_), 2.81 (d, *J* = 16.9, H-3_β_), 1.69 (s, 2-Me); ^13^C NMR (CDCl_3_; 100 MHz) *δ* 198.3 (C-4), 166.0 (C-5), 163.0 (C-6), 161.5 (C-8), 154.8 (C-10a), 144.4 (C-9a), 107.1 (C-5a), 103.8 (C-4a), 102.5 (C-9), 102.1 (C-10), 100.9 (C-2), 97.1 (C-7), 56.1 (6-OMe), 48.3 (C-3), 28.4 (2-Me). |
| 53 | Fonsecin B |  | 305.1010 (M+H)^+^  [305.1019; calculated for C_16_H_17_O_6_;  Δ= ‒3.2 ppm] | ^1^H NMR data in (CD_3_)_2_SO were fully consistent with those reported in literature (Shaaban et al. 2012).  Yellow solid; ^1^H NMR data (CDCl_3_; 400 MHz) *δ* 6.50 (brs, 1H), 6.45 (d, *J* = 2.1, 1H), 6.29 (d, *J* = 2.1, 1H), 3.95 (s, 3H), 3.88 (s, 3H), 3.00 (d, *J* = 16.9, 1H), 2.90 (d, *J* = 16.9, 1H), 1.73 (s, 3H). |
| 54 | Nigragillin |  | 223.1799 (M+H)^+^  [223.1805; calculated for C_13_H_23_N_2_O;  Δ= ‒2.6 ppm] | Only partial NMR data have been reported in literature (Caesar et al. 1969; Isogai et al. 1975). ^1^H and ^13^C NMR data for nigragillin are presented below.  ^1^H NMR data (CD_3_OD; 400 MHz) *δ* 7.19 (dd, *J* = 14.6, 10.8, H-3’), 6.42 (d, *J* = 14.6, H-2’), 6.32 (m, H-4’), 1.85 (m, H-6’), 4.52*^a^* (br m, H-2), 4.00 (br m, H-6_α_), 3.39 (br m, H-6_β_), 2.97 (br m, H-5), 2.80 (dd, *J* = 12.3, 5.2, H-3_α_), 2.47 (dd, *J* = 12.3, 2.9, H-3_β_), 2.84 (s, H_3_-8), 1.85 (d, *J* = 6.6, H_3_-6’), 1.30 (d, *J* = 6.8, H_3_-7), 0.99 (d, *J* = 6.6, H_3_-9); ^13^C NMR (CD_3_OD; 100 MHz) *δ* 168.8 (C-1’), 145.1 (C-3’), 139.2 (C-5’), 131.5 (C-4’), 118.9 (C-2’), 56.1 (C-5), 53.0 (C-3), 48.5 (C-2)*^b^*, 44.5 (C-6) *^b^*, 42.7 (C-8), 18.7 (C-6’), 16.9 (C-7), 8.9 (C-9).  *^a^*Irradiation of the signal at *δ* 4.52 resulted in the simplification of the doublet at *δ* 1.30 to a singlet as well as the resonances at *δ* 2.80 and *δ* 2.47 to doublets.  *^b^*These assignments were made using HMBC correlations from methyl groups. |
| 55 | Carbonarone A |  | 230.0805 (M+H)^+^  [230.0812; calculated for C_13_H_12_NO_3_;  Δ= ‒2.9 ppm] | NMR data were fully consistent with those reported in literature  (Zhang et al. 2007) |
| 56 | *Epi*-pestalamide A |  | 344.1113 (M+H)^+^  [344.1129; calculated for C_18_H_18_NO_6_;  Δ= ‒4.5 ppm] | ^1^H NMR data in (CD_3_)_2_CO were fully consistent with those reported in literature (Ding et al. 2008)  [α]^25^_D_ +15 (*c* 0.10, MeOH); ^1^H NMR data (CDCl_3_; 400 MHz) *δ* 11.72 (br s, NH-15 or COOH-19), 8.72 (s, H-2), 7.19-7.39 (m, H-9 ‒ H-13), 6.30 (s, H-5), 3.88 (s, H_2_-7), 3.23 (dd, *J* = 18.0, 8.4, H_α_-17), 3.02 (m, H-18), 2.86 (dd, *J* = 18.0, 4.9, H_β_-17), 1.27 (d, *J* = 7.3, H_3_-20). |
| 57 | 1,8-dimethoxy-naphthalene |  | 189.0909 (M +H)^+^  [189.0910; calculated for C_12_H_13_O_2_;  Δ= ‒0.6 ppm] | NMR data were fully consistent with those reported in literature  (Yang et al. 2008) |
| 58 | *o*-Methyldihydro-gladiolic acid |  | 207.0643 (M‒CH_3_OH+H)^+^  [207.0652; calculated for C_11_H_11_O_4_;  Δ= ‒4.3 ppm] | NMR data were fully consistent with those reported in literature  (Ichihara et al. 1985) |
| 59 | Altenuene |  | 293.1007 (M+H)^+^  [293.1020; calculated for C_15_H_17_O_6_;  Δ= ‒4.3 ppm] | NMR data were consistent with those reported in literature  (Altemöller et al. 2006) |
| 60 | Monascuskaoliaone B  (New) |  | 299.2213 (M+H)^+^  [299.2217; calculated for C_17_H_31_O_4_;  Δ= ‒1.3 ppm] | NMR data: See the manuscript |
| 61 | Biscognin A  (New) |  | 213.1126 (M+H)^+^  [213.1121; calculated for C_11_H_17_O_4_;  Δ= +2.2 ppm] | NMR data: See the manuscript |
| 62 | Biscognin B  (New) |  | 223.0602 (M+H)^+^  [223.0601; calculated for C_11_H_11_O_5_;  Δ= +0.4 ppm] | NMR data: See the manuscript |

**X**

S2: ^1^H NMR (400 MHz; top) and ^13^C NMR (100 MHz; bottom) spectra of biscognin A (**1**) in CDCl_3_

**X**

S3: ^1^H NMR (400 MHz; top) and ^13^C NMR (100 MHz; bottom) spectra of biscognin B (**2**) in CDCl_3_

**X**

**X**

S4: ^1^H NMR (400 MHz; top) and ^13^C NMR (175 MHz; bottom) spectra of monascuskaoliaone B (**4**) in CDCl_3_

S5: Observed chemical shift differences (Δ*δ* = *δ*_S_ − *δ*_R_, ppm; 500 MHz) for the *R*- and *S*-MTPA

esters of (a) biscognin A (**1**) and (b) monascuskaoliaone B (**4**)

**a**)

| **COMPOUND** | **RESULT** |
| --- | --- |
| Monascuskaoliaone (Mon_*R*)   | No. conformers: **18**  Boltzmann Distribution (coverage): **92.1%**  Energy cut: **2.0 kcal/mol** |
| Monascuskaoliaone (Mon_*S*)   | No. conformers: **33**  Boltzmann Distribution (coverage): **97.8%**  Energy cut: **2.0 kcal/mol** |
| Monascuskaoliaone B (MonB_*RR*)   | No. conformers: **14**  Boltzmann Distribution (coverage): **93.5%**  Energy cut: **2.0 kcal/mol** |
| Monascuskaoliaone B (MonB_*SR*)   | No. conformers: **10**  Boltzmann Distribution (coverage): **91.5%**  Energy cut-off: **2.0 kcal/mol** |

**b**)

**
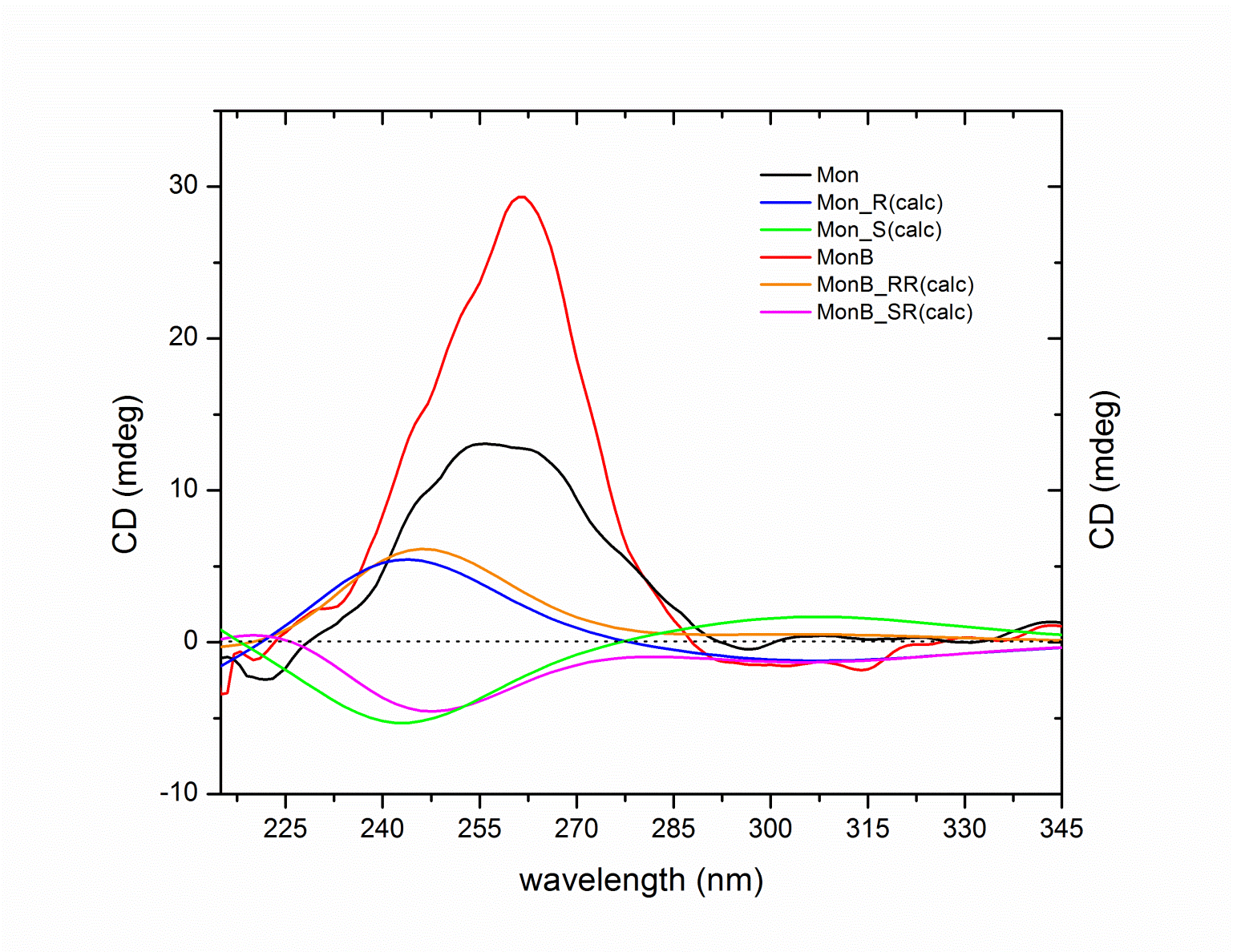
**

**c**)

| **COMPOUND** | **[α]_25_^D^ value (exp)** | **COMPOUND** | **[α]_D_ value (calc.)** |
| --- | --- | --- | --- |
| Monascuskaoliaone | Due to low sample amount, a credible optical rotation value could not be obtained. Instead ECD data were collected for this optically active sample. | Monascuskaoliaone (Mon_2*R*) | +32 |
|  |  | Monascuskaoliaone (Mon_2*S*) | −22 |
| Monascuskaoliaone B | +29.8 (c 0.067, CH_3_OH) | Monascuskaoliaone B (MonB_2*R15R*) | +141 |
|  |  | Monascuskaoliaone B (MonB_2*S15R*) | −141 |

S6: (**a**) Conformational analysis, (**b**) ECD spectra (experimental and calculated), and (**c**) specific rotation values (experimental and calculated) of monascuskaoliaone B (**4**), monascuskaoliaone (**5**), and their corresponding C-2 epimer and enantiomer, respectively, in MeOH using DFT B3LYP/DGDZVP

S7. Phylogram of the most likely tree (-lnL = 5345.50) from a RAxML analysis of 41 isolates based on complete ITS rDNA (436 bp). Numbers refer to RAxML bootstrap support values ≥ 70% based on 1000 replicates. Bar indicates nucleotide substitution per site. For each OTU, the plant tissue type is indicated on the right.


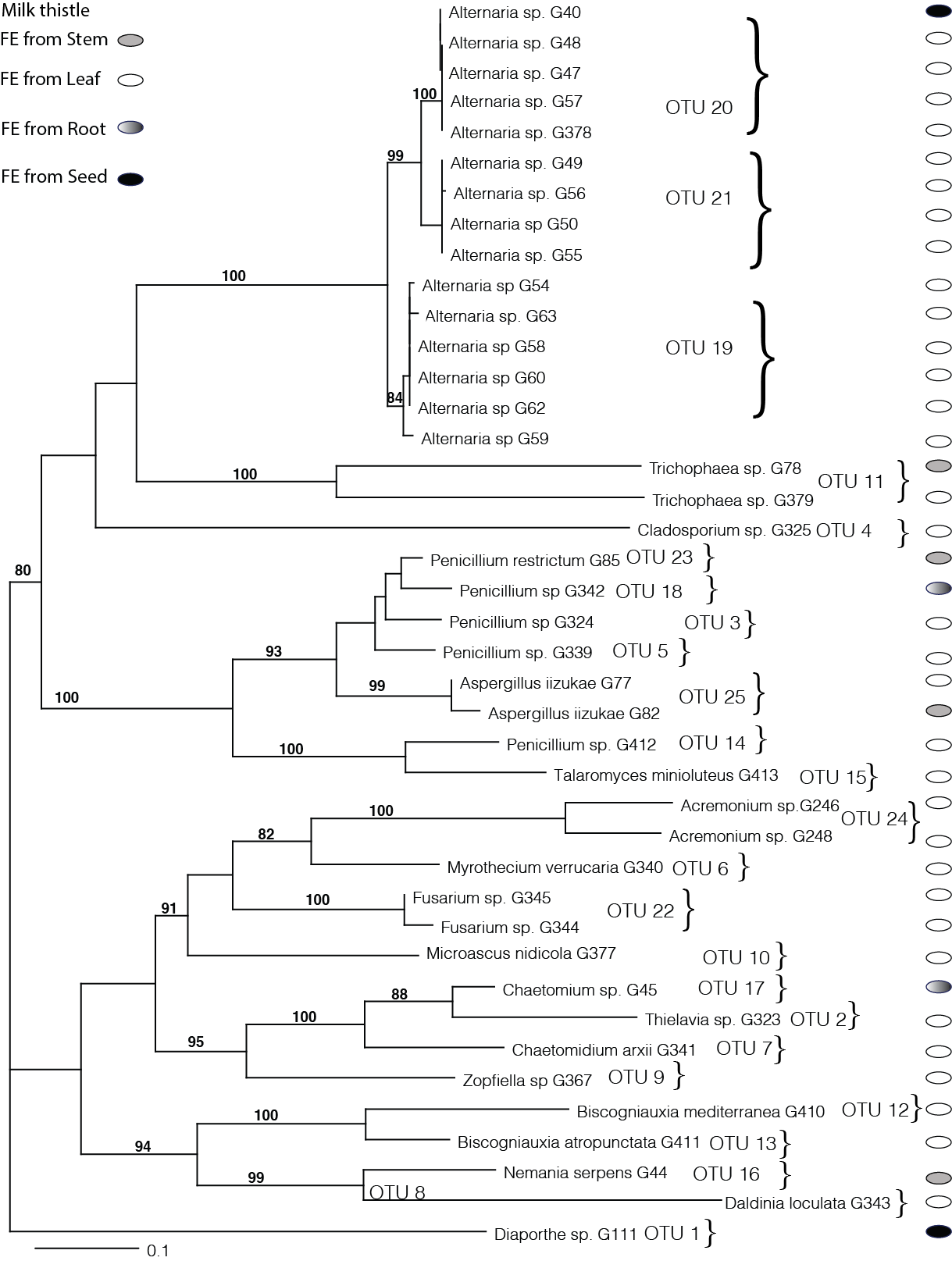


S8. Phylogram of the most likely tree (-lnL = 27826.94) from a RAxML analysis of 226 isolates based on partial LSU rDNA (1264 bp). Numbers refer to RAxML bootstrap support values ≥ 70% based on 1000 replicates. Fungal endophytes from milk thistle are highlighted in bold. Classification following (Lumbsch and Huhndorf 2010) is shown on the right. Bar indicates nucleotide substitution per site.


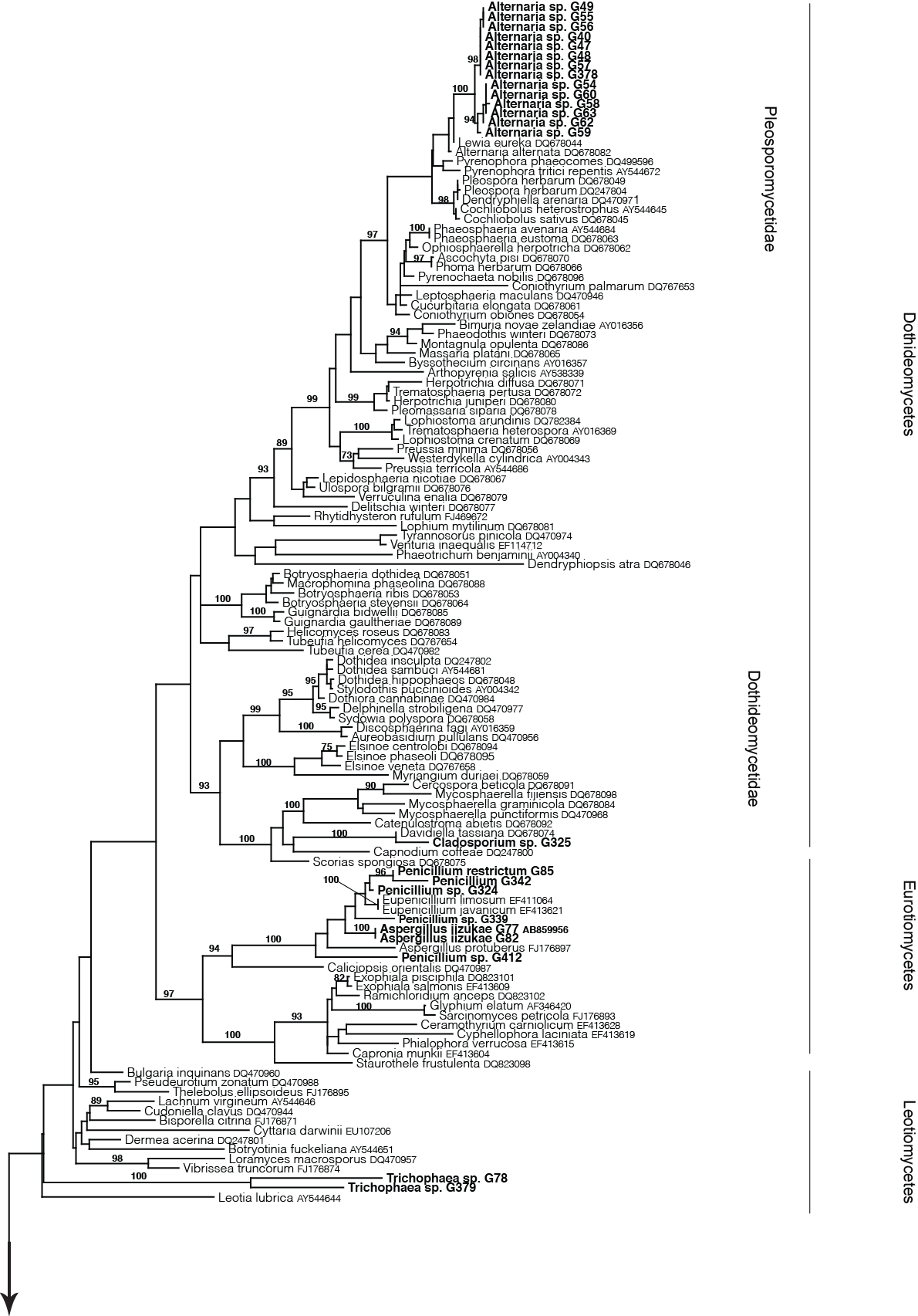


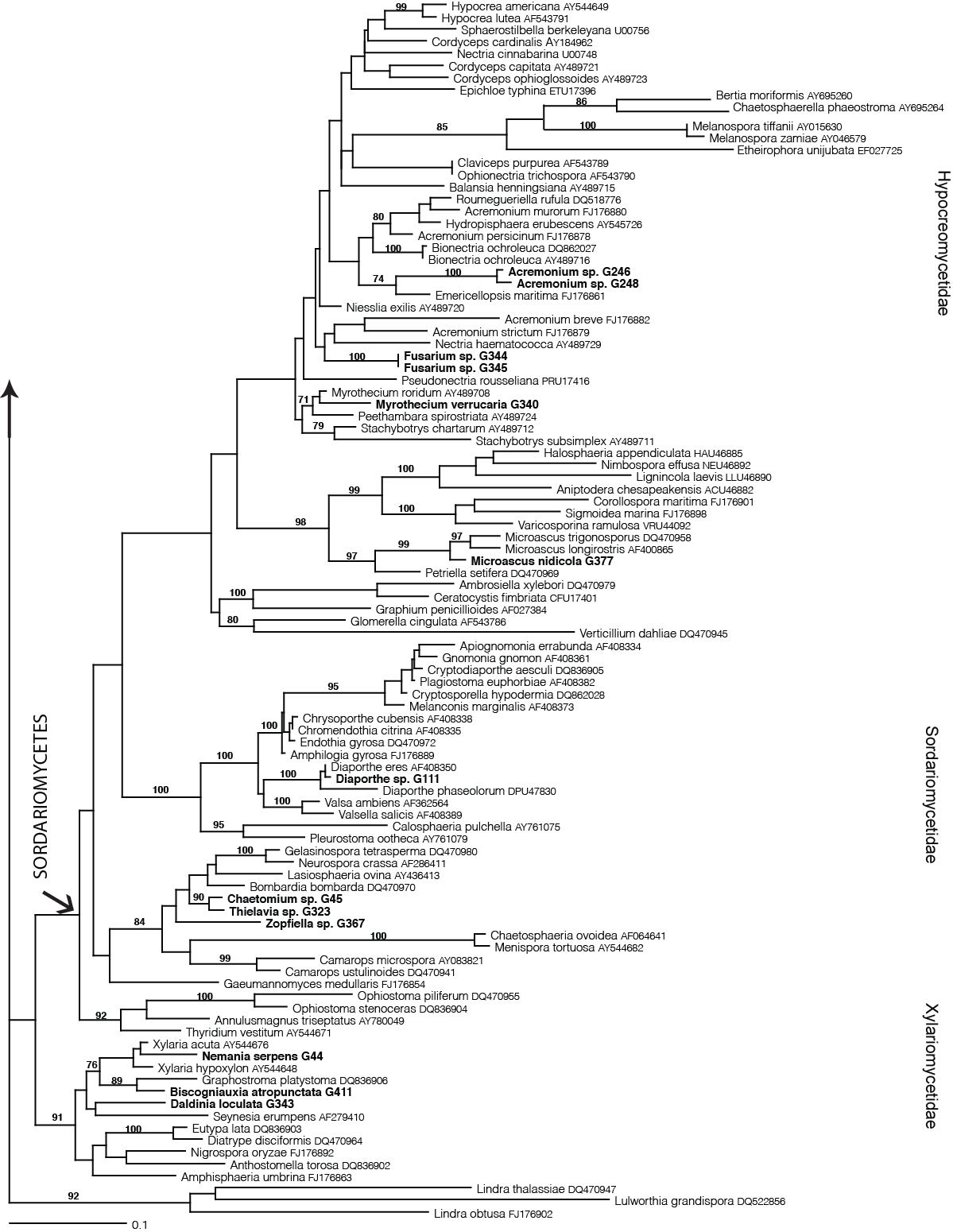


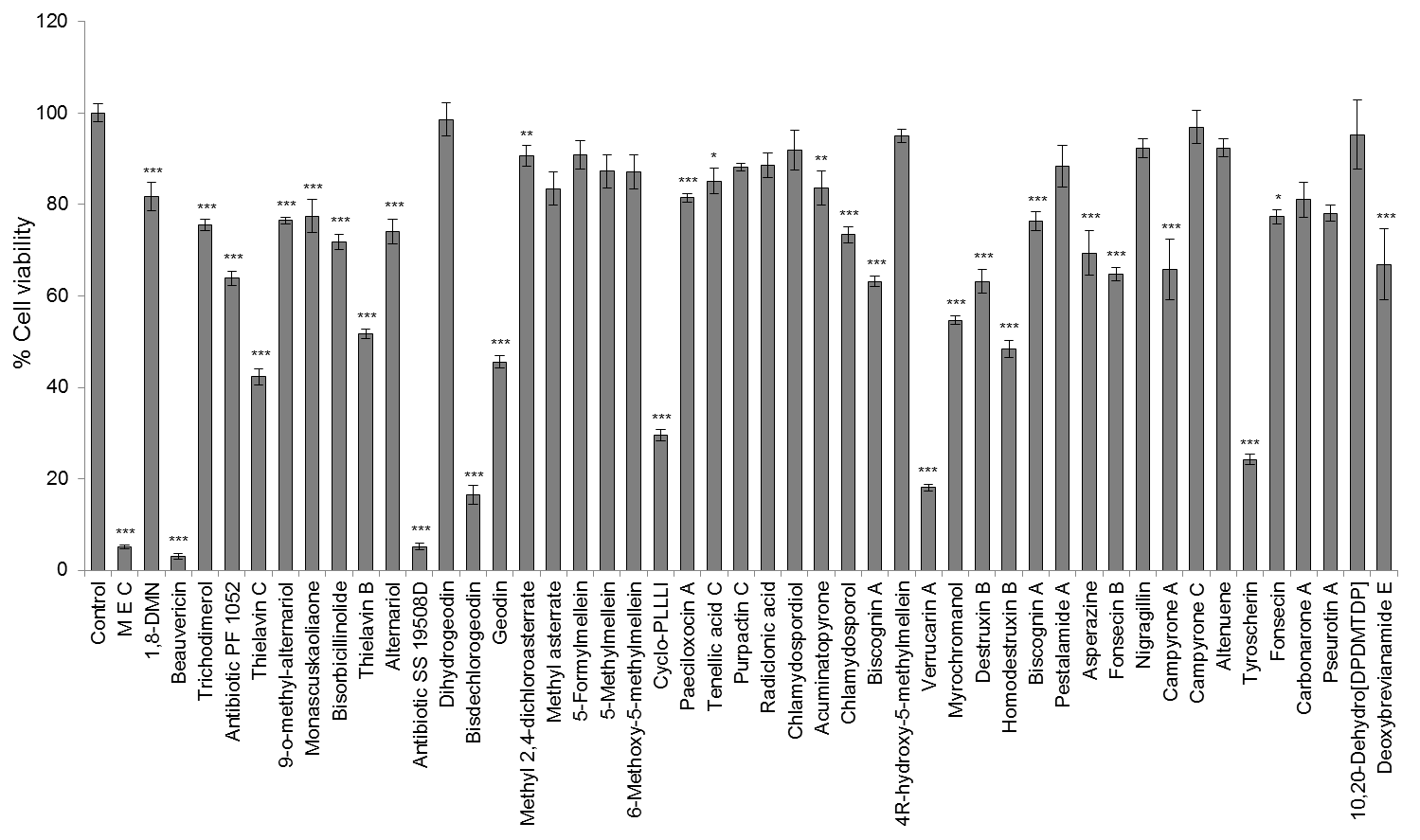


**Epi-**

MEC: Mixture of Euplectin (major):Coneuplectin (minor)

1,8-DMN: 1,8-dimethoxy-naphthalene

Cyclo-PLLLI: Cyclo-[L-Phe-L-Leu-L-Leu-L-Leu-L-Ile]

10,20-Dehydro[DPDMTDP]: 10,20-Dehydro[12,13-dehydroprolyl-2-(1,1-dimethylallyl)tryptophyl]diketopiperazine]

S9. Effect of selected fungal metabolites on the viability of human prostate carcinoma (PC-3) cells

Preparation of Mosher’s Esters of biscognin A (**1**) and monascuskaoliaone B (**4**)

Preparation of Mosher’s Esters of biscognin A (**1**): A solution of **1** (0.1 mg) in CDCl_3_ (100 µl) was treated with pyridine-*d*_6_ (20 µl) and *R*-(−)-α-methoxy-α-(trifluoromethyl)-phenylacetyl chloride (*R*-MTPA-Cl, 20 µl). This mixture was allowed to stand for 24 h at room temperature in a screw-cap vial, resulting in formation of the *S*-MTPA ester. Additional CDCl_3_ (500 µl) was then added directly to the vial, and the resulting solution was placed in an NMR tube for analysis. *S*-MTPA ester: key ^1^H NMR data (400 MHz; CDCl_3_) *δ* 5.17 (dq, *J* = 9.5, 6.2, H-8), 3.01 (m, H-7), 1.67 (s, 5-Me), 1.39 (d, *J* = 6.2, H_3_-9), 1.13 (d, *J* = 7.1, 7-Me). *R*-MTPA ester: key ^1^H NMR data (400 MHz; CDCl_3_) *δ* 5.16 (dq, *J* = 9.7, 6.1, H-8), 3.05 (dq, *J* = 9.7, 7.1, H-7), 1.75 (s, 5-Me), 1.36 (d, *J* = 6.1, H_3_-9), 1.17 (d, *J* = 7.1, 7-Me).

Preparation of Mosher’s esters for monascuskaoliaone B (**4**): A solution of **4** (0.1 mg) in pyridine-*d*_6_ (100 µl) was treated with *R*-MTPA-Cl (10 µl). After this mixture was allowed to stand overnight at room temperature resulting in formation of the *S*-MTPA ester, it was further diluted with pyridine-*d*_6_ (500 µl) and analyzed by NMR. *S*-MTPA ester: key distinguishable ^1^H NMR data (400 MHz; CDCl_3_) *δ* 5.73 (s, H-4), 5.17 (m, H_2_-7), 4.81 (m, H-15), 3.06 (m, H_2_-6), 1.38 (s, 2- Me), 0.89 (t, *J* = 7.4, H_3_-17). *R*-MTPA ester: key ^1^H NMR data (400 MHz; CDCl_3_) *δ* 5.75 (s, H-4), 5.18 (m, H_2_-7), 4.81 (m, H-15), 3.07 (m, H_2_-6), 1.37 (s, 2- Me), 0.77 (t, *J* = 7.4, H_3_-17).

Computational methods

Theoretical calculations of the ECD spectra for monascuskaoliaone B (**4**), monascuskaoliaone (**5**), and their corresponding C-2 epimers/enantiomers were performed using the Gaussian 09 (Gaussian Inc., Pittsburgh, PA, USA) program package ([Frisch et al. 2009](#_ENREF_2)). Geometry optimizations for all compounds were carried out using the PM3 semi-empirical force field calculations as implemented in the Spartan 08 program (Wavefunction Inc. Irvine, CA, USA) ([Kong et al. 2000](#_ENREF_3)). A Monte Carlo search protocol ([Chang et al. 1989](#_ENREF_1)) was carried out considering an energy cutoff of 2 kcal/mol. In each case, the minimum energy structures were filtered and checked for duplicity. Each conformer was geometrically optimized using hybrid DFT method B3LYP and basis set DGDZVP (B3LYP/DGDZVP), and thermochemical parameters and the frequencies at 298 K and 1 atm. The self-consistent reaction field method (SCRF) with conductor-like continuum solvent model (COSMO) was employed to perform the ECD calculation of major conformers for all compounds in methanol solution with the same basis set. The calculated excitation energy (in nm) and rotatory strength R, in dipole velocity (*R*_vel_) and dipole length (*R*_len_) forms, were simulated into an ECD curve by using the following Gaussian function:

$$\Delta\varepsilon\left( E \right)= \sum_{i=1}^{n} \Delta\varepsilon_{i}\left( E \right)= \sum_{i=1}^{n} \left( \frac{R_{i}E_{i}}{2.29 \times{10}^{-39}\sqrt{\pi\sigma}}exp\left[ -\left( \frac{E-E_{i}}{\sigma} \right)^{2} \right] \right)$$

where σ is the width of the band at 1/*e* height, and *E_i_* and *R_i_* are the excitation energies and rotatory strengths for transition *i*, respectively. σ = 0.40 eV and *R_vel_* were used. All quantum calculations were carried out on a Linux operating system in the KanBalam cluster from a Hewlett-Packard HP CP 4000, which includes 1368 AMD Opteron processors at 2.6 GHz and a RAM memory of 3 terabytes (KanBalam, Dirección General de Cómputo y de Tecnologías de Información y Comunicación, UNAM).

Supplementary References

Chang G, Guida WC, Still WC. 1989. An internal-coordinate Monte Carlo method for searching conformational space. J Am Chem Soc 111: 4379–4386. doi:10.1021/ja00194a035

Frisch MJ, Trucks GW, Schlegel HB, Scuseria GE, Robb MA, Cheeseman JR, Scalmani G, Barone V, Mennucci B, Petersson GA, Nakatsuji H, Caricato M, Li X, Hratchian HP, Izmaylov AF, Bloino J, Zheng G, Sonnenberg JL, Hada M, Ehara M, Toyota K, Fukuda R, Hasegawa J, Ishida M, Nakajima T, Honda Y, Kitao O, Nakai H, Vreven T, Montgomery Jr. JA, Peralta JE, Ogliaro F, Bearpark MJ, Heyd J, Brothers EN, Kudin KN, Staroverov VN, Kobayashi R, Normand J, Raghavachari K, Rendell AP, Burant JC, Iyengar SS, Tomasi J, Cossi M, Rega N, Millam NJ, Klene M, Knox JE, Cross JB, Bakken V, Adamo C, Jaramillo J, Gomperts R, Stratmann RE, Yazyev O, Austin AJ, Cammi R, Pomelli C, Ochterski JW, Martin RL, Morokuma K, Zakrzewski VG, Voth GA, Salvador P, Dannenberg JJ, Dapprich S, Daniels AD, Farkas Ö, Foresman JB, Ortiz JV, Cioslowski J, Fox DJ. 2009. Gaussian 09. Gaussian, Inc., Wallingford, CT, USA.

Lumbsch HT, Huhndorf, SM 2010. Outline of Ascomycota – 2009. Fieldiana Life and Earth Sciences 1: 1–60.

Kong J, White CA, Krylov AI, Sherrill D, Adamson RD, Furlani TR, Lee MS, Lee AM,

Gwaltney SR, Adams TR, Ochsenfeld C, Gilbert ATB, Kedziora GS, Rassolov VA, Maurice DR, Nair N, Shao Y, Besley NA, Maslen PE, Dombroski JP, Daschel H, Zhang W, Korambath PP, Baker J, Byrd EFC, Van Voorhis T, Oumi M, Hirata S, Hsu C-P. Ishikawa N, Florian J, Warshel A, Johnson BG, Gill PMW, Head–Gordon M, Pople JA, 2000 Q-Chem 2.0: a high-performance ab initio electronic structure program package. J Comput Chem 21: 1532–1548. doi:10.1002/1096-987X(200012)21:16<1532::AID-JCC10>3.0.CO;2-W
